# Molecular and cellular correlates of human nerve regeneration: *ADCYAP1*/PACAP enhance nerve outgrowth

**DOI:** 10.1093/brain/awaa163

**Published:** 2020-07-11

**Authors:** Georgios Baskozos, Oliver Sandy-Hindmarch, Alex J Clark, Katherine Windsor, Pall Karlsson, Greg A Weir, Lucy A McDermott, Joanna Burchall, Akira Wiberg, Dominic Furniss, David L H Bennett, Annina B Schmid

**Affiliations:** a1 Nuffield Department of Clinical Neurosciences, The University of Oxford, Oxford, UK; a2 Department of Clinical Medicine, The Danish Pain Research Center, Aarhus, Denmark; a3 Institute of Neuroscience and Psychology, College of Medical, Veterinary and Life Sciences, University of Glasgow, Glasgow, UK; a4 Nuffield Department of Orthopaedics, Rheumatology and Musculoskeletal Sciences, The University of Oxford, Oxford, UK

**Keywords:** carpal tunnel syndrome, nerve regeneration, ADCYAP1, pituitary adenylate cyclase-activating peptide, peripheral nerve injury

## Abstract

We only have a rudimentary understanding of the molecular and cellular determinants of nerve regeneration and neuropathic pain in humans. This cohort study uses the most common entrapment neuropathy (carpal tunnel syndrome) as a human model system to prospectively evaluate the cellular and molecular correlates of neural regeneration and its relationship with clinical recovery. In 60 patients undergoing carpal tunnel surgery [36 female, mean age 62.5 (standard deviation 12.2) years], we used quantitative sensory testing and nerve conduction studies to evaluate the function of large and small fibres before and 6 months after surgery. Clinical recovery was assessed with the global rating of change scale and Boston Carpal Tunnel Questionnaire. Twenty healthy participants provided normative data [14 female, mean age 58.0 (standard deviation 12.9) years]. At 6 months post-surgery, we noted significant recovery of median nerve neurophysiological parameters (*P *<* *0.0001) and improvements in quantitative sensory testing measures of both small and large nerve fibre function (*P *<* *0.002). Serial biopsies revealed a partial recovery of intraepidermal nerve fibre density [fibres/mm epidermis pre: 4.20 (2.83), post: 5.35 (3.34), *P *=* *0.001], whose extent correlated with symptom improvement (*r* = 0.389, *P *=* *0.001). In myelinated afferents, nodal length increased postoperatively [pre: 2.03 (0.82), post: 3.03 (1.23), *P *<* *0.0001] suggesting that this is an adaptive phenomenon. Transcriptional profiling of the skin revealed 31 differentially expressed genes following decompression, with *ADCYAP1* (encoding pituitary adenylate cyclase activating peptide, PACAP) being the most strongly upregulated (log2 fold-change 1.87, *P *=* *0.0001) and its expression was associated with recovery of intraepidermal nerve fibres. We found that human induced pluripotent stem cell-derived sensory neurons expressed the receptor for PACAP and that this peptide could significantly enhance axon outgrowth in a dose-dependent manner *in vitro* [neurite length PACAP 1065.0 µm (285.5), vehicle 570.9 μm (181.8), *P *=* *0.003]. In conclusion, carpal tunnel release is associated with significant cutaneous reinnervation, which correlates with the degree of functional improvement and is associated with a transcriptional programme relating to morphogenesis and inflammatory processes. The most highly dysregulated gene *ADCYAP1* (encoding PACAP) was associated with reinnervation and, given that this peptide signals through G-protein coupled receptors, this signalling pathway provides an interesting therapeutic target for human sensory nerve regeneration.

## Teaser

Carpal tunnel release provides an opportunity to study sensory nerve regeneration in humans. Schmid *et al.* show that cutaneous reinnervation correlates with symptom improvement after surgery. *ADCYAP1* (encoding PACAP) expression is associated with sensory reinnervation *in vivo* and facilitates neuron outgrowth *in vitro*.

## Introduction

Knowledge of cellular and molecular correlates of nerve regeneration is critical to understand the temporal profile of nerve regeneration as well as the determinants of successful recovery.

Preclinical data suggest a complex interplay of several pathways in regulating nerve regeneration including retrograde neuronal signalling (e.g. importin proteins), successful Wallerian degeneration, phenotypic changes in Schwann cells (e.g. growth factors, cell adhesion molecules such as neuregulin 1), transcriptional changes in Schwann cells and neurons (e.g. cAMP activated CREB, ATF-3, cJun, microRNA) and local protein translation at the growth cone (e.g. ZBP1 induced β-actin translation) (for review see Scheib and Hoke, 2013).

While neural regeneration has been extensively studied in animal models of peripheral nerve injury (Navarro, 2016; Dubovy *et al.*, 2018), studying the determinants of nerve regeneration and its relationship to recovery in humans remains challenging due to the limited access to tissues and a lack of treatments to promote nerve regeneration. So far, studies have mostly relied on sensory or motor reinnervation times, the advancement of Tinel’s sign as well as the recovery of action potentials in electrophysiological recordings (Seddon *et al.*, 1943; Dolenc and Janko, 1976; Buchthal and Kuhl, 1979). Through these studies it has become evident that human nerve regeneration is less successful compared to regeneration identified in preclinical rodent experiments. This may be attributed to the longer regeneration distances and chronic denervation in humans, which is in contrast to acute regeneration models over short distances commonly studied in preclinical work (Hoke, 2006).

In the past decade, serial skin biopsies have provided insights into the regeneration of small sensory axons in humans (Rajan *et al.*, 2003; Polydefkis *et al.*, 2004; Hahn *et al.*, 2006; Petersen *et al.*, 2010). Recently, skin biopsies have also been used to obtain information about the integrity of myelination and large nerve fibres (Nolano *et al.*, 2003; Schmid *et al.*, 2014). However, a lack of knowledge remains concerning the molecular and cellular determinants of nerve regeneration and their relationship to clinical recovery and specifically neuropathic pain in humans.

Here, we use carpal tunnel syndrome (CTS) as a model system to study sensory nerve regeneration in humans after chronic denervation. CTS is one of the few neuropathic conditions that can be treated surgically. It therefore provides a unique model system to prospectively study the cellular and molecular correlates of nerve regeneration and their relationship to clinical recovery in humans. We deeply phenotyped patients before and 6 months after carpal tunnel decompression including electrophysiology, quantitative sensory testing, symptom profiling and functional deficits. Using serial skin biopsies, we determined the postoperative reinnervation of sensory target tissue with histology as well as the molecular signature associated with regeneration using RNA sequencing. This revealed that *ADCYAP1* was the most differentially expressed gene (DEG). *ADCYAP1* encodes PACAP (pituitary adenylate cyclase-activating peptide), which has pleiotropic functions, including as a neurotrophic factor, neuromodulator and neurotransmitter (Waschek, 2002; Tamas *et al.*, 2012). PACAP binds with high affinity to three receptors (PAC1, VPAC1 and VPAC2). Whereas PAC1 specifically binds PACAP, the VPAC receptors have equal affinity for PACAP and VIP (vasointestinal peptide). We found *ADCYAP1* and its receptors expressed in human induced pluripotent stem cell-derived (hiPSCd) sensory neurons *in vitro* as well as in human skin, with PACAP and PAC1 specifically expressed in human sensory afferents. There is a body of predominantly preclinical evidence implicating *ADCYAP1*/PACAP with neural regeneration (for review see Waschek, 2002) through downstream cAMP signalling (Guirland *et al.*, 2003; Ravni *et al.*, 2008; Emery and Eiden, 2012). We therefore studied the effect of PACAP given to hiPSCd-sensory neurons and found it enhanced neurite outgrowth in a dose-dependent way. A similar regenerative effect of the selective PAC1 agonist (maxadilan) suggests that manipulation of PACAP/PAC1 may have therapeutic potential in human sensory nerve regeneration.

## Materials and methods

### Study design and participants

This study used a prospective longitudinal cohort design. Seventy-three patients with clinically and electrodiagnostically confirmed CTS were recruited from the surgical waiting lists at Oxford University Hospitals NHS Foundation Trust. Patients were excluded if electrodiagnostic testing revealed abnormalities other than CTS, if another medical condition affecting the upper limb and neck was present (e.g. hand osteoarthritis, cervical radiculopathy), if there was a history of significant trauma to the upper limb or neck, or if CTS was related to pregnancy or diabetes. Patients undergoing repeat carpal tunnel surgery were excluded. Whereas 60 patients underwent surgery and were included in the main cohort, 13 patients opted out of surgery after being consented, with the study team having no role in these patients’ decisions. While these patients were not the main focus of the study, we continued to follow them over time and report their data as a separate cohort. A cohort of 20 healthy volunteers (proportionally age- and gender-matched to the CTS surgery group) without any systemic medical condition, or a history of hand, arm or neck symptoms, served as healthy control subjects.

The study was approved by the London Riverside national research ethics committee (Ref 10/H0706/35), and all participants gave informed written consent in accordance with the Declaration of Helsinki. Whereas the healthy participants only attended a single session, all patients attended two appointments, one at baseline and a follow-up appointment (∼6 months after surgery/baseline appointment). In patients with CTS, the hand that was operated (surgery group) or the hand that was more affected (no-surgery group) was tested, whereas the non-dominant hand was tested in healthy controls. All clinical measurements were collected by the same experienced examiner to ensure consistency.

#### Sample size calculation

The required sample size was *a priori* determined with G-Power software (Faul *et al.*, 2007) for the primary outcome measure of small fibre regeneration following surgery using previous data from the cross-sectional Oxford CTS cohort (Schmid *et al.*, 2014). Fifty-nine patients were required to detect a 20% increase in intraepidermal nerve fibre density (IENFD) with 80% power, significance set at α = 0.05 and an effect size of 0.328.

### Clinical phenotype

#### Symptom and function questionnaires

Patients with CTS completed the Boston Carpal Tunnel Questionnaire (symptom and function scale; 0 = no symptoms/disability, 5 = very severe symptoms/disability) (Levine *et al.*, 1993) to determine symptom severity and functional deficits. Clinical recovery was determined with two global rating of change scales (GROC) (Jaeschke *et al.*, 1989) evaluating changes in hand symptoms and function. The GROC scales range from ‘a very great deal better’ (+7), to ‘a very great deal worse’ (−7) with a change of ≥5 (a good deal better/worse) considered clinically meaningful (Kamper, 2009). In addition, we calculated the difference in the Boston questionnaire subscales (follow-up minus baseline).

#### Electrodiagnostic tests

Electrodiagnostic tests were performed with an ADVANCE™ system (NeuroMetrix) for sensory and motor latencies and amplitudes of the median, ulnar and radial nerve as previously described (Schmid *et al.*, 2014) (Supplementary material). Electrodiagnostic testing was graded according to the scale by Bland (2000) ranging from very mild (1), to extremely severe (6). During data analysis, absent sensory and motor recordings were replaced with values of zero for amplitudes but excluded from analysis of latencies and nerve conduction velocities to prevent inflated results.

#### Quantitative sensory testing

#####  

Quantitative sensory testing (QST) was carried out according to the standardized protocol published by the German research network of neuropathic pain (Rolke *et al.*, 2006). Cold and warm detection (CDT, WDT) and cold and heat pain thresholds (CPT, HPT) as well as thermal sensory limen (TSL) were measured with a thermotester (Somedic). We also examined mechanical detection (MDT), vibration detection (VDT), mechanical pain thresholds (MPT), pressure pain thresholds (PPT), wind up ratio (WUR) and mechanical pain sensitivity (MPS). The number of paradoxical heat sensations during TSL and the presence of dynamic mechanical allodynia were quantified. All measurements apart from PPT and VDT were performed over the palmar aspect of the proximal phalanx of the index finger. PPT was recorded over the thenar eminence and VDT over the palmar side of the distal end of the second metacarpal. To achieve normally distributed data, all QST parameters, expect for CPT, HPT and VDT, were log-transformed (Magerl *et al.*, 2010). A small constant of 0.1 was added to MPS to avoid loss of zero-rating values. Most QST measures have good to excellent long-term reliability except for WUR, which has fair retest reliability over a period of 4 months (Marcuzzi *et al.*, 2017; Nothnagel *et al.*, 2017).

### Histological analysis of target tissue

#### Skin biopsies

Two serial skin biopsies (3 mm diameter) were taken 6 months apart on the ventrolateral aspect of the proximal phalanx of the index finger, innervated by the median nerve. The second biopsy was taken a few millimetres more proximal, avoiding the primary biopsy site. One surgical patient received warfarin and no follow-up biopsy was thus taken. The biopsies were performed under sterile conditions following local anaesthesia with 1% lidocaine (1–1.8 ml). Whereas half of the biopsy was snap-frozen for transcriptional analysis (see below), the other half was fixed in fresh periodate-lysine-paraformaldehyde for 30 min before being washed in 0.1 M phosphate buffer and cryoprotected in 15% sucrose in 0.1 M phosphate buffer. The tissue was embedded in OCT, frozen and stored at −80°. To ensure consistency, each histological analysis was carried out by the same blinded observer.

#### Intraepidermal nerve fibre density

Sections (50 μm) were cut on a cryostat and stained using a previously described protocol (Schmid *et al.*, 2014). Serial biopsies of the same patient were processed simultaneously to minimize variability. Sections were blocked with 5% fish gelatine before incubation with the primary antibodies for myelin basic protein (MBP, Abcam, 1:500) and protein gene product 9.5 (PGP, Ultraclone, 1:1000) overnight. Secondary antibodies were also incubated overnight (Alexa 488 1:1000, Abcam; Cy3, Stratech 1:500). The PGP antibody from Ultraclone was discontinued during our study and replaced with the PGP antibody from Zytomed (1:200) after successful benchmarking.

IENFD was established by counting three sections per participant using an Axio LSM 700 microscope (Zeiss) (Lauria *et al.*, 2010). Sections were then photographed at 20× magnification (Observer Z1, Zeiss) and epidermal length was measured with ImageJ (NIH, USA). Average IENFD was expressed as fibres/mm epidermis. Intra-rater reliability to determine IENFD was examined by processing and counting *n *=* *23 skin samples twice, 1 week apart. Intraclass correlation coefficient (ICC) revealed almost perfect intra-rater agreement for IENFD [ICC(2,1) 0.913 (95% confidence interval 0.791–0.963), *P *<* *0.0001].

#### Myelinated fibre integrity

We used several approaches to evaluate myelinated fibre integrity. First, we quantified the number of Meissner corpuscles per mm epidermis as previously reported (Schmid *et al.*, 2014). Second, dermal nerve bundles containing myelinated fibres were expressed as a ratio of total PGP9.5+ nerve bundles containing at least five PGP+ axons per mm^2^ dermis (excluding the subepidermal plexus). Third, we measured the length of nodes of Ranvier in the subepidermal plexus of MBP/PGP double-stained sections in up to 10 sections/participant. This was achieved with the Zen black software (Zeiss) on confocal image stacks taken at ×40 magnification as previously reported (Schmid *et al.*, 2014). Nodes were classified as elongated if they exceeded 6.1 μm in length (Doppler *et al.*, 2013). If fewer than 15 nodes were identified, patients were excluded from the analysis (*n *=* *8).

#### Subepidermal plexus length

We used unbiased stereology to estimate nerve fibre length in the subepidermal plexus using a previously described methodology (Karlsson *et al.*, 2013). Estimation of nerve fibre length in the subepidermal plexus was performed on PGP stained skin sections using an Olympus BX51 microscope (60× oil immersion lens) and newCAST stereological software (Visiopharm) in a blinded fashion. A virtual plane probe was used, as it generates isotropy of the test planes and allows length estimation of tubular objects in thick sections with arbitrary directions. The region of interest was the subepidermal plexus, here defined as an area deep to the epidermal-dermal junction and 200 μm towards the dermis. Papillae were excluded as they contain Meissner corpuscles, which are not part of the subepidermal plexus and their irregular distribution may have biased the results. Briefly, 3D sampling boxes are superimposed over the skin section, thereby generating randomized isotropic virtual planes in systematically sampled fields of view (Karlsson *et al.*, 2013). Sampling box height was set to 15 μm and the box area size to 7908 μm^2^. Sampling steps were 85 × 70 μm (in the *x* and *y*-direction), with a plane separation distance of 25 μm. Four sections were typically counted per patient (ranging from three to six) and nerve fibre length expressed as length per squared area.

### Statistical analysis of clinical and histological data

Data were analysed using SPSS (version 24, IBM) and R software (Team, 2018). Normal distribution of clinical and histological data was established by visual inspection and with the Kolmogorov-Smirnov test for normality.

The main statistical comparison concerned the pre- versus post-surgical data. This was achieved using two-sided paired *t*-tests or Wilcoxon signed rank tests for parametric and non-parametric data, respectively. In order to establish differences at baseline and the extent of recovery, pre- and post-surgical data of patients with CTS were compared with healthy control data using two-sided independent *t*-tests or Mann Whitney U-tests where appropriate. To control for false discovery rate (FDR), Benjamini-Hochberg correction was applied for three comparisons with the false discovery rate set to 5% (Benjamini and Hochberg, 1994). Unadjusted *P*-values are reported for ease of interpretation. Data of non-operated patients were analysed and reported using the same statistical analyses as for operated patients.

#### Correlation of histological findings with changes in symptomatology/function

Spearman’s correlation analyses were performed in the full patient cohort to determine associations between the extent of clinical recovery and neural regeneration. The extent of neural regeneration was determined by calculating the difference (follow-up minus baseline) of the histological parameters, which changed significantly after surgery (IENFD, percentage of elongated nodes, mean nodal length). Clinical recovery was evaluated with the global rating of change questionnaire (for symptoms and function) as well as with the difference in Boston questionnaire (follow-up minus baseline for symptoms and function subscales). Benjamini-Hochberg correction was used to correct for multiple testing (12 correlations), with unadjusted *P*-values reported. To aid interpretation, Boston questionnaire data were multiplied by −1 so that a positive correlation always corresponds with more pronounced clinical recovery.

### Molecular analysis of target tissue

#### RNA sequencing

Half of the skin biopsies of 47 patients with CTS (*n *=* *29 females) before and after surgery were snap-frozen in liquid nitrogen. RNA was extracted using a combination of phenol extraction and column purification. Samples were homogenized in TRIzol™ and mixed with chloroform before spinning for phase separation. The aqueous liquid phase containing the nucleic acids was removed and added to the columns of the High Pure RNA tissue kit (Roche Diagnostics). RNA was purified using repeated wash steps and DNAse treatment. The extracted RNA was sent for sequencing at the Wellcome Trust Centre for Human Genetics in Oxford, UK. All samples were normalized to 650 ng prior to library preparation using the TruSeq Stranded Total RNA Library Prep Kit (Illumina) and poly-A enrichment. Paired-end sequencing was performed using the HiSeq4000 platform with a read length of 75 bp.

Reads were mapped to the GRC.h.38 Human genome using STAR, gene counts were generated using HTSeq and DEG analysis was performed using DESeq2 as outlined in detail in the Supplementary material. Gene ontology enrichment for biological processes for DEGs was carried out using topGO (Alexa and Rahnenfuhrer, 2018) and GSEA (Morgan *et al.*, 2017).

#### Gene to regenerative phenotype association

Fold gene expression changes relative to baseline [(post-surgery – pre-surgery) / pre-surgery] and individual baseline expression of significant DEGs were tested for association with fold changes and post- versus pre- differences in IENFD and nodal length. This was achieved by testing significant Pearson correlation coefficients and by binning the continuous outcomes into four categories corresponding to the four quarters, as defined by the outcome quartiles with significance assessed using one-way ANOVA. In all associations of gene expression or fold change to phenotype, significance cut-off was an FDR-adjusted *P*-value < 0.05.

#### Droplet digital PCR to validate RNA sequencing

The findings of the RNA sequencing were validated in 78 paired samples pre- and post-surgery (*n *=* *39 patients) using droplet digital PCR (ddPCR). Complementary DNA was prepared using the EvoScript Universal cDNA Master kit (Roche Diagnostics). TaqMan™ assays (Thermo-Fisher) for *ADCYAP1* (Hs00174950_m1) and *HPRT1* (Hs03929098_m1) were run in duplicates with 1 μl of target probe labelled with FAM added to 1 μl of reference probe labelled with VIC in the same reaction using the ddPCR Supermix for Probes (Bio-Rad). QuantaSoft v1.7.4.0917 software (Bio-Rad Laboratories) was used to extract data (copies/μl) and normalized gene expression was reported (ratios of target over reference data).

#### Localization of PACAP and PAC1 in skin biopsies

The localization of PACAP (encoded by *ADCYAP1*) expression in skin biopsies was evaluated using an immunofluorescent staining protocol adapted from Hannibal *et al.* (1995). In brief, 50-μm skin sections were mounted on glass slides and incubated in 10% goat serum in phosphate-buffered saline (PBS) and Triton^TM^ X-100 for 30 min. Primary antibodies for PACAP (gift from Prof Jan Fahrenkrug, 1:5) and PGP9.5 (Zytomed, 1:200) were incubated overnight.

A biotinylated goat anti-mouse antibody (Vector Laboratories, 1:200) was then applied for 2 h along with an Alexa Fluor^®^ 546 anti-rabbit antibody (Life Technologies, 1:500). After five washes, the sections were incubated for 30 min with an avidin-biotin-horseradish peroxidase (VECTASTAIN^®^ Elite ABC Kit, Vector Laboratories) before washing four times. The sections were incubated for 10 min in FITC conjugated tyramide (Perkin Elmer, 1:100) diluted in 0.1 M borate buffer containing 0.0003% hydrogen perioxide. After three washes, slides were coverslipped in mounting medium and imaged on an Observer Z1 confocal imaging system (Zeiss) using the same settings within each patient (pre and post-surgery). Whereas the specificity of the PACAP antibody has been extensively evaluated (Hannibal *et al.*, 1995), we included a negative control (omission of primary antibody) as well as a pre-adsorption control. We quantified average PACAP staining intensity of *n *=* *10 patients before and after surgery using ImageJ (NIH USA) within (i) an area overlying the epidermis and subepidermal plexus including both neuronal and non-neuronal structures; and (ii) specifically within PGP+ nerve fibres using thresholding.

Further, potential PAC1 localization in human skin afferents was determined by double immunostaining with PAC1 (Cambridge Bioscience, 1:100) and PGP (Bio-Rad, 1:200). In brief, 50 μm skin sections were heat treated for 5 min in 10 mM citric acid with 0.05% Tween 20 followed by 30 min incubation in 10% goat serum in PBS and Triton^TM^ X-100 and primary antibody incubation overnight. The next day, sections were washed and incubated with biotinylated goat anti-rabbit antibody (Vector Laboratories, 1:200) for 2 h followed by Alexa Fluor^®^ 546 anti-mouse antibody (Life Technologies, 1:1000) and Streptavidin (Life Technologies, 1:500) for 2 h. Sections were then mounted and imaged on an Observer Z1 confocal imaging system (Zeiss).

### *In vitro* expression and role of ADCYAP1/PACAP

#### RNA-sequencing of human induced pluripotent stem cell-derived sensory neurons

We interrogated our data from a previous RNA sequencing experiment (Baskozos *et al*., 2019) in hiPSCd sensory neurons, which replicate many of the molecular, morphological and functional features of native human sensory neurons (Chambers *et al.*, 2012; Clark *et al.*, 2017; McDermott *et al.*, 2019). The hiPSCd sensory neurons were generated as previously described (Clark *et al.*, 2017). RNA was extracted with the hybrid method of combined phenol extraction (TriPure, Roche) and column purification (High Pure RNA tissue Kit, Roche). Sequencing was performed using the Illumina HiSeq4000 paired-end protocol with 75-bp reads. Reads were mapped on the Hg38 (GRCh38) human genome using STAR with the default ENCODE options. Gene counts were calculated for the GRCh38.88 gene set annotation using HTSeq. Data are available in the GEO series GSE107181. We evaluated *ADCYAP1* and its primary receptor *PAC1* as well as secondary receptors *VPAC1* and *VPAC2* with affinity for PACAP and VIP (vasoactive intestinal protein) expression levels compared to other genes.

#### Differentiation of human induced pluripotent stem cell-derived sensory neurons

We used two control iPSC lines derived from fibroblasts of a healthy 44-year-old female (Subject NHDF) (Hartfield *et al.*, 2014) and a healthy 51-year-old male (Subject AD2) (NRES Committee South Central – Berkshire, UK, REC 10/H0505/71). The lines were differentiated to sensory neurons as previously described (Chambers *et al.*, 2012; Clark *et al.*, 2017; Weir *et al.*, 2017) and as outlined in the Supplementary material.

#### Human sensory neuron regeneration model

To determine a potential regenerative role of PACAP in human primary sensory neurons, we dissociated mature hiPSCd sensory neurons and assessed their ability to regenerate neurites, as we have previously described (McDermott *et al.*, 2019). Neurons were enzymatically treated for 30 min with 0.1% Trypsin (ThermoFisher), followed by mechanical dissociation with a fire polished glass pipette. Single cells were replated onto Matrigel™ treated coverslips at low density in the presence of different doses of PACAP protein (10 nM to 10 μM, AnaSpec and Bachem), vehicle (0.01% DMSO, Sigma) or the selective PAC1 agonist maxadilan (1 μM, Bachem) before fixation and analysis by immunocytochemistry 18 h later.

#### Quantification of neurite outgrowth

Three differentiations of mature hiPSCd sensory neurons were used (27 ± 3 weeks old). Neurons were taken from individual wells of a 24-well plate and dissociated separately to form each experimental unit (*n*). Four dissociation experiments were performed. Average neurite outgrowth was calculated from 6–10 technical replicates (individual coverslips of re-plated neurons) per *n* (for *n* in each experiment, refer to single datapoints in graphs). The vehicle- and PACAP-treated hiPSCd sensory neurons were immunostained with NF200 using immunohistochemistry. NF200 has been shown to be expressed in virtually all DRG neurons in humans (Chang *et al.*, 2018). The NF200 antibody (Sigma, 1:500) was added to hiPSCd sensory neurons overnight at room temperature. Neurons were then washed three times with PBS + Triton^TM^ X-100 and a secondary anti-mouse antibody Alexa Fluor^®^ 488 (Life Technologies: 1:1000) was added for 2 h at room temperature. The neurons were washed three times with PBS + Triton^TM^ X-100 and the coverslips were mounted with VECTASHIELD^®^ on slides and sealed.

To determine neurite outgrowth, 20× magnified images of individual hiPSCd sensory neurons were taken on an Observer Z1 imaging system (Zeiss) and analysed for neurite length using WIS-Neuromath software (Rishal *et al.*, 2013). Only neurons with a neurite at least half the diameter of the cell body were included. Neurite length was determined in micrometre per cell and the resultant neurite length from individual neurons was averaged per coverslip [median number of neurons/dissociation 49 (interquartile range, IQR 73)]. Independent *t*-tests or one-way ANOVA followed by Fisher’s least significant difference (LSD) *post hoc* tests were used to compare neurite length among conditions.

Neuronal binding of PACAP (biotinylated protein, AnaSpec) and localization of PAC1 (Cambridge Bioscience, 1:50) in hiPSCd sensory neurons was examined by double-labelling with NF200 (Abcam, 1:2000) using the immunohistochemistry protocol outlined above.

### Data availability

The data that support the findings of this study are deposited in the dbGaP repository (https://www.ncbi.nlm.nih.gov/projects/gap/cgi-bin/study.cgi?study_id=phs001796.v1.p1).

## Results

### The majority of patients show significant symptomatic and functional improvement

Sixty patients with electrodiagnostically confirmed CTS [mean age 62.5 (standard deviation, SD 12.2), 36 females] were examined before and 6 months after decompression surgery. Twenty healthy volunteers served as healthy controls [mean age 58.0 (12.9), 14 females] (Table 1). Post-surgically, 83.3% of patients rated their symptoms at least ‘a good deal better’ (≥5) on the global rating of change scale. None of the patients experienced a deterioration of symptoms. In relation to hand function, 63.3% of operated patients felt at least ‘a good deal better’. The self-perceived symptoms and function improvements were consistent with a postoperative decrease of the Boston Carpal Tunnel Questionnaire symptom and function subscales [Boston symptoms mean (SD) pre: 2.8 (0.7), post: 1.5 (0.5), *t*(59) = 13.8, *P *<* *0.0001; Boston function pre: 2.2 (0.8), post: 1.5 (0.5), *t*(59) = 7.00, *P *<* *0.0001]. However, symptoms did not fully resolve in all patients, and 46.6% continued to have some pain and 38.3% to feel paraesthesia or numbness, although these residual symptoms were mostly mild in nature.

**Table 1:** Demographic and clinical data of patients with carpal tunnel syndrome and healthy volunteers

	Surgery	No-surgery	Controls	*P*-value
Number of participants	60	13	20	
Gender, female/male (% female)	36/24 (60.0)	9/4 (69.2)	14/6 (70.0)	0.649^#^
Mean age, years (SD)	62.5 (12.2)	53.6 (11.9)*	58.0 (12.9)	0.044
Mean weight, kg (SD)	74.4 (15.0)	74.1 (13.1)	73.6 (15.7)	0.978
Mean height, cm (SD)	168.4 (9.5)	165.2 (8.8)	167.0 (7.6)	0.472
Mean symptom duration, months (SD)	64.2 (96.5)	52.7 (44.8)		0.676
Mean Boston scale (SD)				
Symptoms	2.8 (0.7)	2.3 (0.6)*		0.008
Function	2.2 (0.8)	1.9 (0.6)		0.184

Comparisons were done with one-way ANOVA and independent *t*-tests.

# Comparisons were done with chi-squared test.

* Statistical significance compared to surgery group.

NPSI = neuropathic pain symptom inventory; SD = standard deviation.

### Electrodiagnostic test severity improves following surgery, but fails to reach normal levels in most patients

Electrodiagnostic testing demonstrated moderate severity preoperatively [median Bland scale 3.0 (IQR 2.8)]. After surgery, electrodiagnostic test severity improved by one grade to an average of mild severity [2.0 (2.0), *z*(55) = −5.62, *P *<* *0.0001] (Table 2). With the exception of eight patients (4.8%), in whom electrodiagnostic testing was within normal limits following surgery, all patients continued to show some degree of median nerve conduction slowing.

**Table 2:** Median nerve neurophysiology data of patients with CTS pre- and post-surgery

	Surgery group			Healthy controls		
	Pre	Post	*P*-value	Baseline	*P*-value (HC-pre)	*P*-value (HC-post)
SNAP, μV	2.8 [8.2]	6.0 [5.5]	**0.004**	13.5 [11.1]	**<0.0001**	**<0.0001**
Sensory NCV, m/s	34.5 [7.9]	41.5 [6.15]	**<0.0001**	50.2 [6.9]	**<0.0001**	**<0.0001**
Distal motor latency, ms	5.4 [1.8]	4.3 [1.0]	**<0.0001**	3.1 [0.7]	**<0.0001**	**<0.0001**
CMAP, mV	4.1 [4.1]	5.2 [4.7]	0.299	7.7 [2.9]	**0.001**	**0.005**
Delay ulnar-median	1.6 [1.5]	0.8 [0.4]	**<0.0001**	0.2 [0.2]	**<0.0001**	**<0.0001**

Data are presented as median [IQR]. CMAP = compound motor action potential; delay ulnar-median = delay between the motor potentials for dorsal interossei (ulnar nerve) and lumbricals (median nerve) measured over an 8 cm wrist segment; HC = healthy controls; NCV = nerve conduction velocity; SNAP = sensory nerve action potential.

*P*-values that were significant following Benjamini-Hochberg correction are highlighted in bold.

### Sensory detection thresholds improve following surgery, but fail to reach normal levels

**Figure 1:**
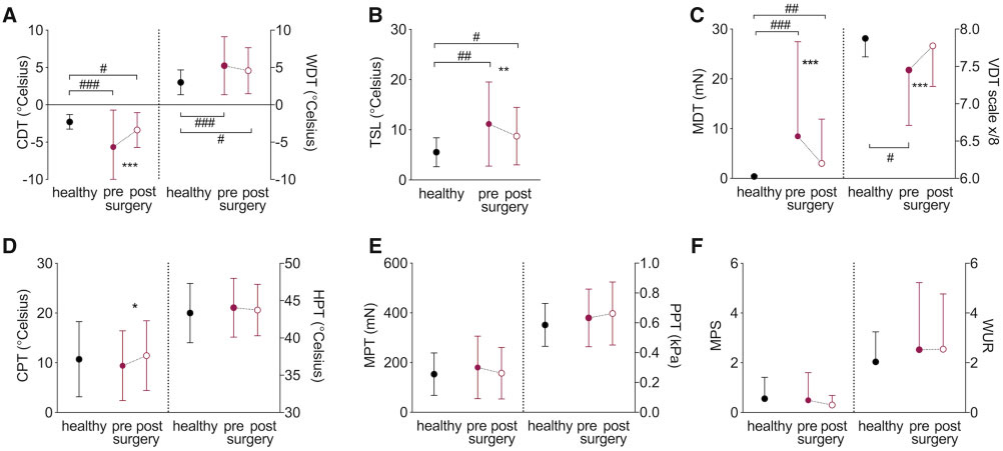
**Recovery of somatosensory function.** Raw quantitative sensory testing data are presented as mean and standard deviations for healthy participants (*n *=* *20, black circles) and patients with CTS before (closed red circle) and after surgery (*n *=* *60, open red circle). (**A**) Cold (CDT) and warm detection thresholds (WDT); (**B**) thermal sensory limen (TSL); (**C**) mechanical (MDT) and vibration detection thresholds (VDT); (**D**) cold (CPT) and heat pain thresholds (HPT); (**E**) mechanical (MPT) and pressure pain thresholds (PPT); (**F**) mechanical pain sensitivity (MPS) and wind-up ratio (WUR). Significant differences for paired/independent *t*-tests after Benjamini Hochberg correction are indicated between groups with ^#^*P *<* *0.05, ^##^*P *<* *0.01, ^###^*P *<* *0.0001 and within groups with *P <* *0.05, **P <* *0.01, ***P <* *0.0001.

Somatosensory function and its recovery measured with quantitative sensory testing in the median innervated territory of the hand can be found in Table 3 and Fig. 1A–C. Patients with CTS had a significant pre-surgical deficit in all thermal and mechanical detection thresholds compared to healthy control subjects [*t*(78) > 2.30, *P *<* *0.015]. Cold detection, thermal sensory limen, mechanical and vibration detection improved following surgery [*t*(59) > 3.20, *P *<* *0.002]. Only warm detection thresholds remained unchanged post-surgery [*t*(59) = 1.58, *P *=* *0.120]. Postoperative detection thresholds, however, mostly failed to reach levels found in healthy control participants [*t*(75) > 2.00, *P *<* *0.033].

**Table 3:** Quantitative sensory testing data of patients pre- and 6 months post-surgery as well as healthy controls

	Surgery group			Healthy controls		
	Pre	Post	*P*-value (pre post)	Baseline	*P*-value (HC-pre)	*P*-value (HC-post)
CDT, °C	−5.66 (4.95)	−3.38 (2.33)	**<0.0001**	−2.28 (0.97)	**<0.0001**	**0.033**
WDT, °C	5.25 (3.89)	4.40 (3.24)	0.120	3.01 (1.66)	**<0.0001**	**0.022**
TSL, °C	11.15 (8.38)	8.74 (5.73)	**0.002**	5.53 (2.89)	**0.002**	**0.023**
MDT, mN	8.45 (19.00)	3.01 (8.90)	**<0.0001**	0.38 (0.26)	**<0.0001**	**0.006**
VDT, x/8	7.45 (0.74)	7.78 (0.54)	**<0.0001**	7.88 (0.32)	**0.015**	0.429
CPT, °C	9.42 (7.00)	11.44 (7.01)	**0.046**	7.88 (0.25)	0.484	0.698
HPT, °C	44.06 (3.94)	43.74 (3.45)	0.481	10.72 (7.56)	0.484	0.664
MPT, mN	180.4 (125.7)	157.2 (103.3)	0.264	153.44 (85.33)	0.657	0.357
MPS, 0–100	0.59 (1.11)	0.41 (0.39)	0.569	0.56 (1.00)	0.782	0.386
PPT, kPa	180.4 (125.7)	157.3 (103.3)	0.221	351.36 (86.22)	0.747	0.452
WUR, ratio	2.53 (2.70)	2.53 (2.24)	0.810	2.04 (1.21)	0.402	0.302

Data are presented as mean (SD) for untransformed data (CPT, HPT, VDT) and retransformed mean for log-tranformed data. *P*-values reflect statistics carried out on log-transformed data as appropriate comparing pre- and postoperative data (pre post), healthy controls with CTS patients pre-surgery (HC-pre) and healthy controls with CTS patients post-surgery (HC-post). *P*-values that were significant following Benjamini-Hochberg correction are highlighted in bold. CDT = cold detection threshold; CPT = cold pain threshold; HPT = heat pain threshold; MDT = mechanical detection threshold; MPS = mechanical pain sensitivity; MPT = mechanical pain threshold; PPT = pressure pain threshold; TSL = thermal sensory limen; VDT = vibration detection threshold; WDT = warm detection threshold; WUR = wind-up ratio.

Thermal and mechanical pain thresholds were not different between healthy controls and preoperative CTS patients (*P *>* *0.402, Fig. 1D–F) and remained largely unaltered following surgery (*P *>* *0.221). The only exception was an increased sensitivity to cold pain following surgery compared to before surgery [*t*(59) = −2.04, *P *=* *0.046]. Of four patients with preoperative paradoxical heat sensations, one patient continued to paradoxically feel heat on cooling postoperatively and two patients experienced new paradoxical heat sensations postoperatively. No patient presented with dynamic mechanical allodynia pre- or postoperatively.

### Intraepidermal nerve fibres partially regenerate following surgery

**Figure 2:**
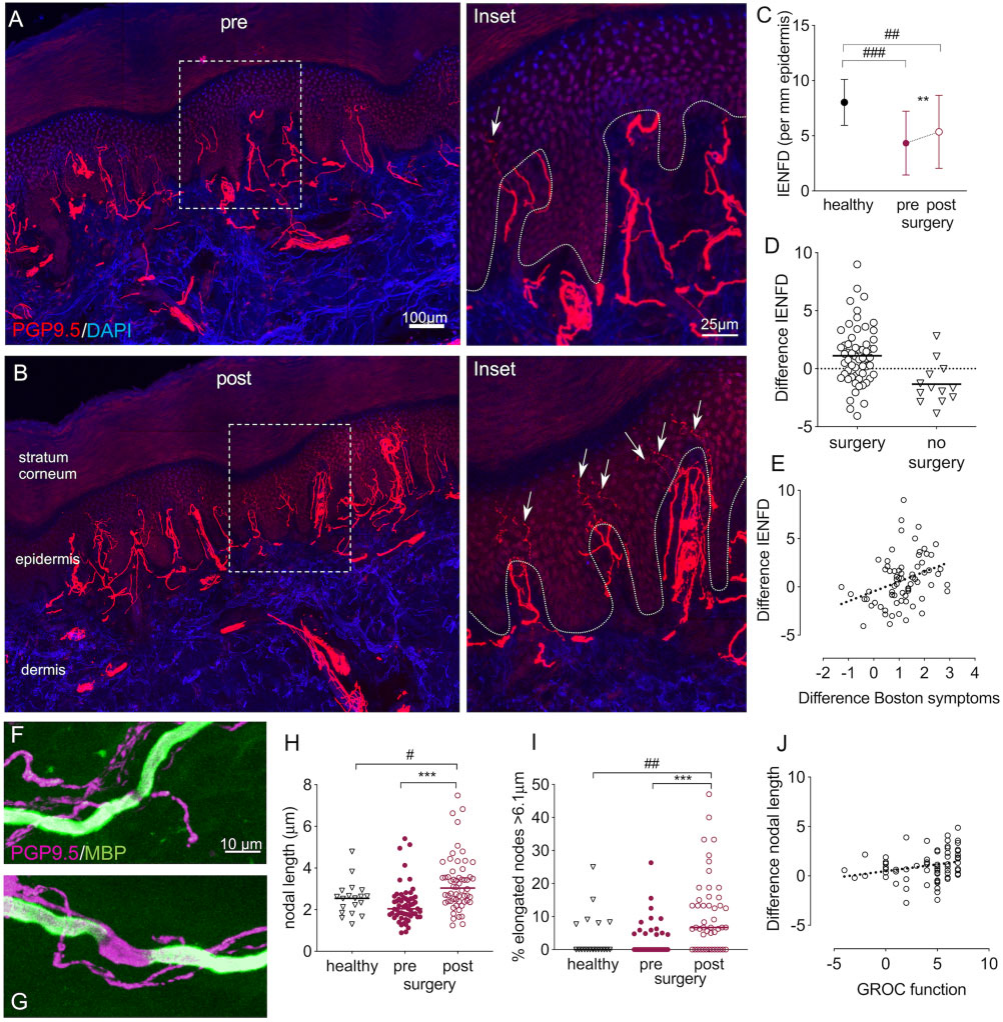
**Histological evidence nerve regeneration in target tissue.** Immunohistochemically stained sections of index finger skin of a patient with CTS before (**A**) and 6 months after surgery (**B**). Cell nuclei are apparent in blue (DAPI) and axons in red (PGP9.5). After surgery, an increased number of small fibres (arrows on ×4 magnified regions of interest on the *right*) penetrate the dermal-epidermal border (dotted line). (**C**) Quantification revealed a reduced IENFD in patients with CTS compared to healthy controls, which increased after surgery but failed to reach normal levels. (**D**) The difference in IENFD post- compared to pre-surgery demonstrated a high interindividual variability, with a continuing small fibre degeneration in patients who were not operated on. (**E**) A more pronounced intraepidermal nerve fibre regeneration (difference IENFD) correlates with improved symptoms (*r* = 0.389, *P *=* *0.001, difference Boston symptom scale data multiplied with −1). (**F**–**J**) Changes in nodal architecture. Example of normal (**F**) and elongated node (**G**) in skin sections stained immunohistochemically with PGP9.5 and myelin basic protein (MBP). (**H**) Median nodal length and (**I**) the median percentage of elongated nodes >6.1 μm increase following surgery and remain higher than healthy controls. (**J**) The correlation between difference in mean nodal length and the global rating of change scale (GROC) for hand function marginally fails to reach significance after Benjamini Hochberg correction (*r* = 0.316, *P *=* *0.008). Significant differences for paired/independent *t*-tests after Benjamini Hochberg correction are indicated between groups with ^#^*P *<* *0.05, ^##^*P *<* *0.01, ^###^*P *<* *0.0001 and within groups with **P <* *0.01, ***P <* *0.0001. Group differences based on *n *=* *59 patients with CTS and *n *=* *20 healthy control subjects.

We next evaluated structural integrity of sensory target tissue innervation. Skin biopsies in the median nerve innervated territory of the hand showed a substantial structural degeneration of small axons in the epidermal layer of patients’ skin compared to healthy controls [fibres/mm epidermis in CTS preoperative mean (SD): 4.20 (2.83); healthy controls: 8.03 (2.08), *t*(77) = 5.3, *P *<* *0.0001] (Fig. 2A–C). Following surgery, IENFD improved [5.35 (3.34), *t*(57) = −3.5, *P *=* *0.001], but failed to reach normal innervation levels [*t*(77) = 3.4, *P *=* *0.001]. Of note, postoperative regeneration was highly variable (Fig. 2D).

### Subepidermal plexus nerve fibre length density remains unchanged

Given the incomplete epidermal reinnervation following surgery, we hypothesized that regenerating axons may reach the dermis but their penetration through the dermal-epidermal junction may be delayed (Navarro *et al.*, 1997; Rajan *et al.*, 2003). However, evaluation of nerve fibre length density in the subepidermal plexus did not reveal any differences in patients pre- and post-surgery [median mm^−^^2^ (IQR) pre: 70.25 (48.98); post: 75.85 (63.95), *z*(57) = 1.07, *P *=* *0.285] (Supplementary Fig. 1A).

### Nodal architecture changes following surgery

To evaluate changes in myelinated fibres, we examined nodal architecture, which was previously found altered in patients with CTS (Schmid *et al.*, 2014) (Fig. 2F–J). At baseline, the median nodal length [healthy: 2.54 IQR (0.98); CTS: 2.03 (0.82), *z*(77) = −1.70, *P *=* *0.088] (Fig. 2H) and the percentage of elongated nodes > 6.1 μm [healthy: 0.00 (7.92); CTS: 0.00 (4.76), *z*(66) = −0.30, *P *=* *0.767] (Fig. 2I) were comparable in patients with CTS and healthy controls. Following surgery, median nodal length [3.03 (1.23), *z*(56) = 4.36, *P *<* *0.0001] and the percentage of elongated nodes increased in patients [post: 6.66 (15.79), *z*(46) = 4.10, *P *<* *0.0001] and remained higher than healthy controls [*z*(77) = 2.58, *P *=* *0.010 and *z*(66) = 2.85, *P *=* *0.004, respectively] (Fig. 2F–I).

The density of Meissner corpuscles, the number of PGP+ axon bundles per mm^2^ dermis and the ratio of dermal PGP+ axon bundles containing myelin remained unchanged at baseline and after surgery (Supplementary Fig. 1B–D).

### Deterioration in small fibre integrity in patients not undergoing surgery

A small number of patients (*n *=* *13) opted not to undergo surgery. These were slightly younger and had less severe symptoms at baseline compared to those undergoing surgery (Table 1). Of note, non-operated patients demonstrated a continuing degeneration of their IENFD over time [mean baseline: 6.94 (SD 3.09); follow-up: 5.61 (3.41), *t*(12) = 2.66, *P *=* *0.021] (Fig. 2D). Data for the other clinical and histological measures of non-operated patients are summarized in Table 4.

**Table 4:** Clinical, quantitative sensory testing and histological outcomes in CTS patients who did not undergo surgery

	Baseline	Follow-up	*P*-value
Questionnaires			
Mean VAS (SD)			
Pain	2.3 (3.0)	1.1 (1.5)	0.268
Numbness	3.8 (3.2)	1.6 (2.2)	0.063
Paraesthesia	3.9 (2.8)	2.6 (3.0)	0.271
Mean Boston scale (SD)			
Symptoms	2.3 (0.6)	2.0 (0.6)	0.215
Function	1.9 (0.6)	1.6 (0.5)	0.171
Mean NPSI (SD)	9.7 (7.7)	5.6 (5.8)	0.186
**Quantitative sensory testing**			
CDT, °C	−3.40 (1.83)	−3.23 (1.65)	0.805
WDT, °C	4.07 (2.39)	3.96 (1.98)	0.914
TSL, °C	6.53 (2.72)	6.90 (3.73)	0.874
MDT, mN	1.27 (1.48)	1.10 (1.16)	0.980
VDT, x/8	7.44 (0.63)	7.28 (1.15)	0.443
CPT, °C	15.31 (8.06)	11.38 (8.68)	**0.022**
HPT, °C	41.12 (2.19)	42.54 (3.00)	0.135
MPT, mN	100.5 (66.9)	111.4 (92.7)	0.565
MPS, 0–100	0.95 (0.84)	0.56 (0.59)	**0.042**
PPT, kPa	325.5 (97.3)	356.4 (109.7)	0.069
WUR, ratio	1.64 (0.66)	2.24 (0.81)	**0.033**
**Histological parameters**			
IENFD	6.94 (3.09)	5.61 (3.41)	**0.021**
Meissner corpuscle density	0.47 (0.31)	0.20 (0.24)	**0.010**
Subepidermal plexus nerve fibre length	137.0 (176.5)	132.6 (110.1)	0.433
PGP+ dermal axon bundles	5.74 (4.20)	4.42 (1.98)	0.299
Ratio of PGP+ dermal bundles containing MBP	1.45 (0.42)	1.57 (0.60)	0.710
Nodal length, median [IQR]	2.00 [1.01]	2.72 [2.63]	**0.023**
% Elongated nodes, median [IQR]	0.00 [4.55]	6.66 [22.22]	0.063
Internodal length	82.8 (25.88)	70.24 (16.83)	0.062
G-ratio	0.84 (0.07)	0.89 (0.06)	0.107

Data are presented as mean (SD) unless indicated otherwise.

CDT = cold detection threshold; CPT = cold pain threshold; HPT = heat pain threshold; IENFD = intraepidermal nerve fibre density (per mm epidermis); MDT = mechanical detection threshold; MPS = mechanical pain sensitivity; MPT = mechanical pain threshold; PPT = pressure pain threshold; TSL = thermal sensory limen; VDT = vibration detection threshold; WDT = warm detection threshold; WUR = wind-up ratio.

### Neural regeneration correlates with symptom improvement

The extent of small fibre regeneration positively correlated with symptom improvement as determined with the Boston symptom questionnaire [*r*(70) = 0.389, *P *=* *0.001] (Fig. 2E). A postoperative increase in nodal length [*r*(67) = 0.302, *P *=* *0.012] (Fig. 2J) and percentage of elongated nodes with GROC for hand function marginally failed to reach significance after Benjamini Hochberg correction [*r*(56) = 0.306, *P *=* *0.019]. None of the other correlations were significant (*P *>* *0.144).

### Thirty-one genes, including *ADCYAP1*, are differentially expressed following surgery

**Figure 3:**
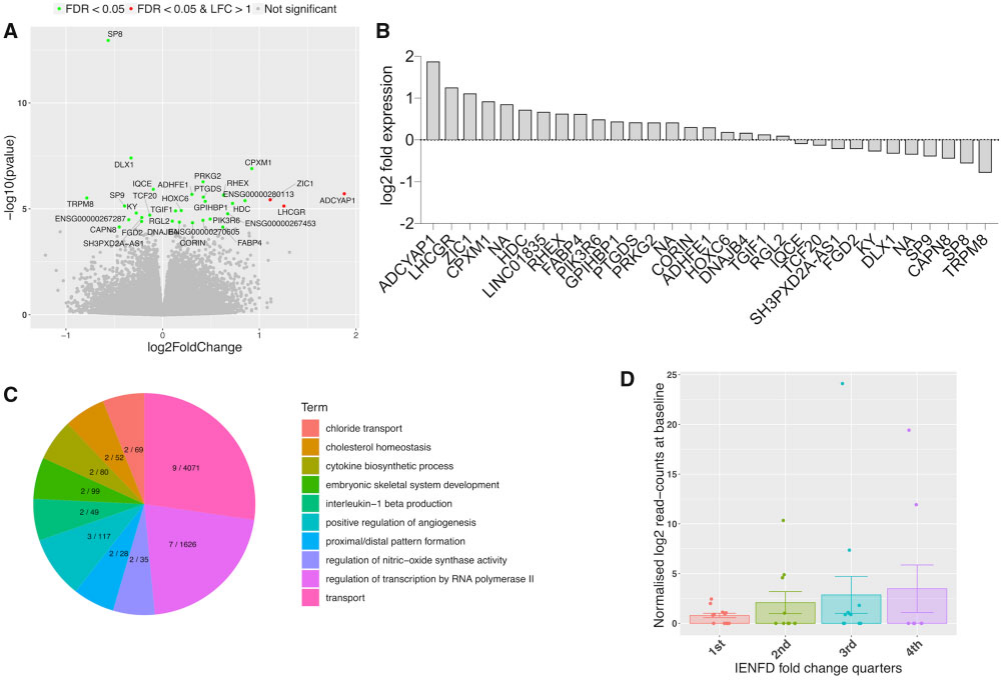
**Molecular signature for nerve regeneration in skin.** (**A**) Volcano plot showing the range of transcriptional changes following carpal tunnel surgery compared to before surgery; *x*-axis log2 (fold change), *y*-axis −log10 (*P*-value). Significant DEGs according to FDR adjusted *P*-value are highlighted and labelled. *ADCYAP1*, i.e. the gene encoding PACAP, was the most upregulated gene. (**B**) Gene expression changes (log2 fold change) for DEGs. (**C**) Top 20 biological process gene ontology (GO) terms from GO enrichment analysis with number of DEGs of total genes marked for each term on the pie chart. (**D**) *ADCYAP1* is upregulated at baseline in all IENFD fold change quarters compared to the lowest quarter of patients with negative IENFD fold change, who continue to degenerate after surgery. Graph shows mean normalized log2 read counts at baseline, standard errors and single datapoints.

To determine a molecular signature associated with nerve regeneration, we performed RNA sequencing of the skin of 47 patients with CTS (*n *=* *29 females) before and after surgical decompression. RNA sequencing revealed 31 significant DEGs in CTS patients post- versus pre-surgery (Fig. 3A and B, Supplementary Fig. 2 and Supplementary Table 1). Gene ontology enrichment analysis for biological processes highlighted biological processes relating to morphogenesis (such as proximal/distal pattern formation, angiogenesis, embryonic limb morphogenesis) and inflammation (such as interleukin 1-beta production and cytokine biosynthetic process) (Fig. 3C and Supplementary Table 2). The most significantly dysregulated gene was *ADCYAP1* (log2 fold change = 1.87, FDR *P*-value = 0.0001) (Fig. 3A and B). *ADCYAP1* encodes PACAP, which is a highly evolutionary conserved protein that is involved in a range of physiological processes including neuronal survival after injury and neurite outgrowth (Waschek, 2002). We confirmed the increased expression of *ADCYAP1* in skin samples following surgical release using ddPCR (Supplementary Fig. 3).

### Expression of DEGs is associated with histological evidence for nerve regeneration

We did not identify FDR-adjusted significant correlations on the individual level between the RNA sequencing determined fold change of the 31 significant DEGs and (i) the fold change; or (ii) differences in IENFD and mean nodal length post-surgery versus pre-surgery.

We also defined four phenotypic groups (quarters) based on the continuous IENFD and nodal length fold change quartiles and tested for significant associations of these groups with baseline DEG expression. Whereas no relationship was detected for nodal length, this analysis revealed that IENFD fold change quarters were significantly dependent on *ADCYAP1*, *DLX1*, *PRKG2*, *ADHFE1*, and *PIK361* baseline expression (*P *<* *0.05). *ADCYAP1* (Fig. 3D) and *ADHFE1* are upregulated in all IENFD fold change quarters compared to the lowest quarter of patients with negative IENFD fold change, i.e. those who continue to degenerate. *PRKG2* is upregulated and *DLX1* and *PIK3R6* are downregulated in patients belonging to the third and fourth quarter.

### PACAP is expressed in human skin and facilitates neurite outgrowth *in vitro*

**Figure 4:**
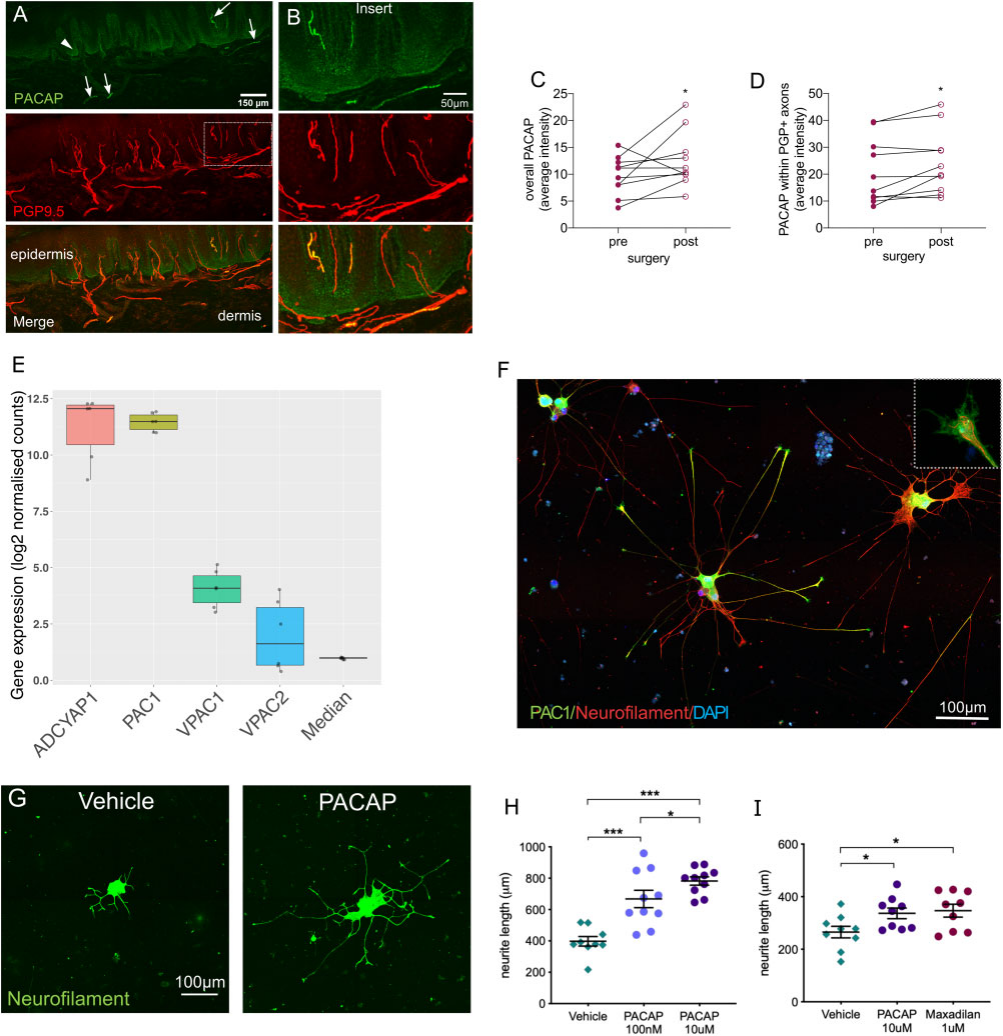
**PACAP is expressed in human skin and induces neurite outgrowth in hiPSCd sensory neurons.** (**A**) Immunohistochemical staining in human finger skin demonstrating presence of PACAP (green) within sensory nerve fibres (PGP9.5, arrows) and in the deeper epidermis (arrowhead). (**B**) Enlarged images of PACAP and PGP9.5 co-localization within sensory afferents. Quantification of PACAP staining intensity reveals an increase in intensity post-surgery compared to baseline within (**C**) an area including epidermal and subepidermal plexus and (**D**) PGP9.5^+^ sensory neurons. *P <* *0.047. (**E**) Gene expression strength of *ADCYAP1*, its main receptor *PAC1* as well as secondary receptors *VPAC1* and *VPAC2* and the median expression of all genes in hiPSCd sensory neurons. The *y*-axis shows RNA-seq log2 normalized gene counts. Box plots show the median and interquartile range with single data-points depicting each experiment (*n *=* *3 cell lines, two differentiations each). (**F**) Immunohistochemical staining of hiPSCd sensory neurons demonstrating expression of the PACAP receptor (PAC1, green) in the soma as well as at the growth cones (×5 magnified *inset*). (**G** and **H**) PACAP given to dissociated mature (27 ± 3 weeks old) and replated hiPSCd sensory neurons enhances neurite outgrowth compared to vehicle in a dose-dependent manner (NHDF line). (**I**) The selective PAC1 agonist maxadilan induces a similar regenerative effect on hiPSCd sensory neurons as PACAP (AD2 line, 27 ± 3 weeks old). Data are presented as mean, standard error of the mean (SEM) and single data-points, *P <* *0.05, **P <* *0.01, ***P <* *0.0001.

We confirmed the localization of PACAP staining within the deeper epidermis close to the basement membrane as well as within sensory afferents in human skin using immunohistochemistry (Fig. 4A and B and Supplementary Fig. 4 for antibody specificity). Quantification confirmed an increase in PACAP protein levels (intensity) after surgery compared to baseline. Of note, this was the case for both PACAP within an area of interest over the epidermis and subepidermal plexus [including the basement membrane: median pre: 10.25 (IQR 5.15); post: 10.76 (5.78), *z *=* *47, *P *=* *0.047] (Fig. 4C) as well as PACAP specifically within sensory neurons [pre: 16.35 (21.00), post: 21.32 (19.00), *z *=* *50, *P *=* *0.022] (Fig. 4D). The PACAP receptors (PAC1, VPAC1, VPAC2) are also present in human skin, as evidenced by mRNA expression as well as weak PAC1 staining within some skin sensory afferents (Supplementary Fig. 5).

Additionally, hiPSCd sensory neurons demonstrated strong mRNA expression of both *ADCYAP1*, as well as its high affinity receptor *PAC1* (Fig. 4E). The mRNA of the secondary receptors *VPAC1* and *VPAC2* are also expressed, albeit at lower levels. Immunostaining for PAC1 revealed that this was predominantly located in cell bodies and growth cones (Fig. 4F), consistent with a role in promoting axon outgrowth. Application of PACAP to hiPSCd sensory neurons increased total neurite outgrowth length in a dose dependent manner compared to vehicle [mean (SD) vehicle: 397.6 μm (90.5); 100 nM PACAP: 667.6 μm (175.1); 10 μM PACAP: 782.0 μm (83.8), *F*(2,26) = 23.45, *P *<* *0.0001, NHDF line] (Fig. 4G, H and Supplementary Fig. 6 for PACAP binding), confirming a proregenerative role of PACAP in human sensory neurons. We replicated these findings in another differentiation of the same cell line as well as another cell line (AD2, Supplementary Fig. 7). The selective PAC1 agonist maxadilan had a similar regenerative effect on hiPSCd sensory neurons as PACAP [mean (SD) vehicle: 265.2 μm (66.2); 10 μM PACAP: 336.5 μm (61.3); 1 μM maxadilan: 346.9 μm (73.12), *F*(2,24) = 3.96, *P *=* *0.03, AD2 line] (Fig. 4I).

## Discussion

Little is known about the molecular and cellular determinants of nerve regeneration in humans. Using CTS as a model system, we demonstrate recovery of large and small fibre function following surgical nerve decompression; however, this recovery remains incomplete even 6 months after surgery. There was significant individual variation in the degree of cutaneous reinnervation, and at group level this did not reach the level seen in unaffected control individuals. We identified a significant correlation between both cutaneous reinnervation by small fibres and the nodal length of myelinated sensory fibres, with symptom and functional recovery following carpal tunnel decompression, respectively. Gene expression analysis identified 31 DEGs, with *ADCYAP1* encoding PACAP being most strongly upregulated. Intriguingly, *ADCYAP1* expression was associated with a histological regenerative phenotype and a regenerative role of PACAP and the PAC1 agonist maxadilan was confirmed in hiPSCd sensory neurons.

Carpal tunnel surgery greatly improved symptoms in the majority of patients (83%), which is in line with previous reports of excellent results in ∼75% of patients (Bland, 2007). The subjective improvement was accompanied by a significant improvement in somatosensory function for all detection thresholds except warm detection. A selectively impaired postoperative recovery of warm detection has previously been reported in patients with entrapment neuropathies including CTS (Nygaard *et al.*, 1996) and lumbar radiculopathy (Tschugg *et al.*, 2016). While this may be attributed to more subtle deficits in warm than cold detection in CTS (Westerman and Delaney, 1991), it may also be explained by a previously observed delayed functional recovery of unmyelinated C-fibres (encoding warm sensations) compared to A-fibres (Lang *et al.*, 1995; Gorodetskaya *et al.*, 2009).

At 6 months after surgery, there was a significant reinnervation of the skin by epidermal nerve fibres; however, this remained incomplete. Given the relatively short reinnervation distance and the previously reported rate of axon regeneration (Fugleholm *et al.*, 1994), one may have expected a higher rate of reinnervation. However, this slow regeneration of small epidermal fibres is in line with previous preclinical work (McLachlan *et al.*, 1994; Lindenlaub and Sommer, 2002) and experimental human studies (Polydefkis *et al.*, 2004; Petersen and Rowbotham, 2010). It has been suggested that the epidermal layer of the skin may be a relatively hostile environment for regenerating fibres as demonstrated by incomplete epidermal regeneration while dermal reinnervation is successful (Navarro *et al.*, 1997; Rajan *et al.*, 2003). Here, we could not find a postoperative increase in the subepidermal plexus length or number of dermal axon bundles. The available quantification methods may, however, not be sensitive to detect relatively subtle changes in dermal small fibres amidst the abundance of large fibres. Alternatively, dermal reinnervation may indeed remain incomplete or only reach subdermal levels, as previously shown for SP+ axons (small peptidergic fibres) following experimental nerve injury (McLachlan *et al.*, 1994). The slow rate of reinnervation emphasizes the point that any future trials of agents to promote nerve regeneration in which epidermal nerve fibres are used as an outcome measure would need to be of significant duration.

Meissner corpuscle and dermal myelinated bundle density in CTS patients were comparable to controls at baseline and remained unaltered after surgery despite a clear improvement in large fibre function assessed by QST (mechanical and vibration detection thresholds). Presumably, the large fibre dysfunction in CTS is driven by ischaemic and demyelinating changes at the lesion site—as has been shown in experimental (Schmid *et al.*, 2013) and clinical nerve compression (Mackinnon *et al.*, 1986)—rather than axon degeneration and changes to target tissue receptors (Schmid *et al.*, 2013, 2014). This is supported by the significant, albeit incomplete recovery of electrophysiological properties over the affected wrist segment following surgical decompression. Persistence of thin myelin sheets after remyelination (Gupta and Steward, 2003; Duncan *et al.*, 2017) may account for the continuing conduction slowing.

Unlike in a previous cohort (Schmid *et al.*, 2014), patients with CTS did not have an increased nodal length at baseline. Since the presence of elongated nodes seems to be protective in nature (Schmid *et al.*, 2014), this disparity may be caused by the inclusion of patients with more severe symptoms in this surgical cohort (not showing protective nodal elongation) compared to the patients with milder symptomatology in the previous cohort (Schmid *et al.*, 2014). Of note, nodal length significantly increased following surgery, further corroborating a protective effect. The mechanism underlying the presence of elongated nodes remains speculative, but it could be associated with the dynamic process of demyelination or remyelination (Suzuki *et al.*, 1969). Indeed, increased nodal length can be found in patients with demyelinating neuropathies (Doppler *et al.*, 2013). Given the association with decreased symptoms in our first cohort and the increased presence of elongated nodes after surgery and their correlation with functional recovery in the present cohort, an association with remyelination in patients with CTS seems more likely. Alternatively, it has been demonstrated that the incorporation of cytoskeletal and ion channel components to nodes of Ranvier requires vesicular transport (Zhang *et al.*, 2012). It is therefore possible that the restoration of axon transport following carpal tunnel surgery enhances delivery of nodal components and nodal lengthening. Elongated nodes may also reflect nerve fibre lengthening during nerve regeneration, as has been shown in experimental limb lengthening in the rabbit (Kerns *et al.*, 2001). The axon and its associated Schwann cell are closely linked at the septate-like junction of the paranode. Lengthening may therefore be more easily achieved at the node rather than at the internode, which would require increased length of both axon and Schwann cell.

We identified a large degree of interindividual variation in the extent of small fibre regeneration and nodal changes, which correlate with clinical recovery. Currently, therapeutic targets that promote nerve regeneration in humans are lacking. Using CTS as a model system, we hypothesized that we could identify a transcriptional signature in the skin that would show a relationship to successful reinnervation by sensory axons. We identified *ADCYAP1* as the most upregulated gene (3.7-fold change) using RNA-seq. We confirmed this upregulation at mRNA level with droplet digital PCR and at protein level with immunostaining. The increased mRNA expression was associated with histological evidence of neural regeneration. *ADCYAP1* encodes PACAP, which is a pleiotropic secreted molecule that has been associated with cell survival in neurodegenerative conditions (Bonaventura *et al.*, 2018) as well as growth cone guidance (Lioudyno *et al.*, 1998) and increased neurite outgrowth of peripheral afferent neurons in preclinical models (Lioudyno *et al.*, 1998; Suarez *et al.*, 2006; Fukiage *et al.*, 2007; Nakajima *et al.*, 2013).

Immunohistochemical studies in cadavers have shown that PACAP is expressed in trigeminal ganglia neurons (Frederiksen *et al.*, 2018) as well as small-to-intermediate sized DRG neurons and the superficial dorsal horn of the spinal cord (Dun *et al.*, 1996), consistent with expression in sensory afferents. Equally, animal studies have shown that PACAP is expressed in small-to-medium sized DRG neurons (Moller *et al.*, 1993) and upregulated during the regenerative process following nerve injury (Zhang *et al.*, 1995, 1996; Woodley *et al.*, 2019), including nerve compression (Pettersson *et al.*, 2004). In relation to localization, we could identify PACAP in sensory afferents and the deeper epidermis in human skin. Whereas future studies are required to determine the source of this PACAP, it may be expressed by keratinocytes (Fig. 4), released and subsequently bound to its receptor PAC1 (which we found was expressed on sensory skin afferents as well as the growth cones of hiPSCd sensory neurons). Indeed, human keratinocytes have previously been shown to express low levels of *ADCYAP1* mRNA (Granoth *et al.*, 2000) and may therefore have a paracrine function. Alternatively, we also found that *ADCYAP1* mRNA is expressed by hiPSCd sensory neurons and therefore may have an autocrine action on these neurons, as previously reported in injured vagus nerves (Reimer *et al.*, 1999).

Whereas a regenerative function of PACAP on sensory neurons has been demonstrated for central (Guirland *et al.*, 2003; Ogata *et al.*, 2015) and peripheral neurons (Suarez *et al.*, 2006; Wu *et al.*, 2006; Nakajima *et al.*, 2013) in animals, its effects on neurite outgrowth in human neurons has not previously been tested. One study reported that PACAP can rescue hiPSCd motor neurons from apoptosis (Bonaventura *et al.*, 2018). We found that administration of PACAP to mature hiPSCd sensory neurons resulted in a dose-dependent increase in neurite outgrowth *in vitro*, thus confirming a direct regenerative action of PACAP on human sensory neurons. PACAP has also been shown to facilitate nerve regeneration through its action on Schwann cells and regulation of the inflammatory response by macrophages (Woodley *et al.*, 2019). These mechanisms may further contribute to the regenerative phenotype associated with PACAP *in vivo*. Intriguingly, the selective PAC1 agonist maxadilan had a similar regenerative effect on hiPSCd sensory neurons, suggesting that manipulation of PACAP/PAC1 may have therapeutic potential in human sensory nerve regeneration.

Whereas we concentrated on *ADACYAP1* as it was the most strongly DEG, its expression correlated with cutaneous reinnervation and had a plausible biological function, several of the other DEGs have been implicated with neurogenesis or neuronal survival. For instance, SP8 and SP9 regulate interneuron development in the olfactory bulb (Li *et al.*, 2018), ZIC1 has been implicated with neural development (Aruga, 2004), DLX1 is necessary for survival of developing retinal ganglion cells (de Melo *et al.*, 2005) and periostin expressed by *POSTN* plays an essential role in axon regeneration following spinal cord injury (Shih *et al.*, 2014). *DNAJB4* is another interesting candidate as it is axonally transcribed (Gomes *et al.*, 2014). Of note, a number of DEGs were also significantly associated with cutaneous reinnervation in patients (e.g. *DLX1*, *PRKG2*, *ADHFE1*, *LINC01835*, *PIK361*). Our dataset can therefore be further exploited to identify biomarkers of and therapeutic targets relating to neural regeneration in humans.

Our unbiased RNA sequencing approach did not identify neurotrophins, which are implicated with skin innervation in preclinical models (Truzzi *et al.*, 2011). Closer inspection of the expression levels of the neurotrophin family in our experiment confirmed that levels were unchanged when comparing before and after surgery (Supplementary Fig. 8), thus excluding a potential concealment of changes due to stringent corrections for multiple testing. The absence of differential expression of neurotrophins in our study may be attributed to several factors. First, the heterogeneity of cells within the skin may dilute potential changes in neurotrophin expression within specific cell types (e.g. keratinocytes versus fibroblasts or immune cells). Second, the molecular signature involved in regeneration following chronic partial denervation (such as in CTS) may not be comparable with the regenerative response modelled in preclinical studies, which mostly use acute denervations. The clinical literature related to neurotrophin levels in the skin of patients with neuropathies is controversial. Whereas some studies report a decrease in neutrophin levels (Anand *et al.*, 1996; Uceyler *et al.*, 2015), others found increased levels (Kennedy *et al.*, 1998; Diemel *et al.*, 1999) compared to healthy controls. Of note, a recent study reported higher levels of neurotrophin family mRNA in patients with diabetic neuropathy following pancreas/kidney transplantation compared to diabetic patients and healthy controls (Saudek *et al.*, 2018). In accordance with our findings however, serial skin evaluation before and after glucose homeostasis did not reveal changes in neurotrophin expression profiles. Also, neurotrophin expression was not related to regeneration of peripheral sensory fibres. Importantly, our work focused on gene expression and we can therefore not make inferences about protein levels. Future work is required to further elucidate a potential role of neurotrophins in human nerve regeneration.

### Limitations of the study and future directions

Because of the ethical consideration of not withholding treatment, and given that our primary aim was to determine nerve regeneration and its relationship to functional recovery, our study used a prospective cohort rather than a trial design of randomizing patients to surgical intervention or conservative management. Nevertheless, we continued to follow-up 13 patients who decided not to undergo surgery. The small sample and the absence of randomization with lower age and symptom severity in non-operated patients precludes a direct comparison. However, the progressive structural decline of small nerve fibres identified in this group is intriguing, and the potential implications for management of these patients warrants further investigation.

We have performed the RNA-seq experiment in skin sections containing a range of cellular components of both neural and non-neural origin. The exact tissue types contributing to the differential expression of *ADCYAP1* therefore remains unknown. Whereas future work is required to determine the potential source of PACAP, our experiments suggest that it can promote axon outgrowth in a human sensory neuronal model.

While we have shown that PACAP is significantly upregulated following cutaneous reinnervation and can promote axon outgrowth in a human sensory neuronal model, determining the exact downstream signalling pathway of PACAP/PAC1-induced neurite outgrowth was beyond the scope of this manuscript, but would be an interesting avenue for future research. Previous experimental work suggests that PACAP induced neurite outgrowth is cAMP-dependent (Guirland *et al.*, 2003; Ravni *et al.*, 2008; Emery and Eiden, 2012) with at least two distinct pathways involved, one of which is PKA-dependent/ERK-independent and the other PKA-independent/ERK-dependent (Emery and Eiden, 2012). Given the multimodal actions of cAMP, targeting upstream at PACAP/PAC1 as a treatment to promote neural regeneration *in vivo* may be more promising. Exploitation of PACAP will however require consideration of optimal delivery given that systemic treatment can trigger migraine (Guo *et al.*, 2016). In fact, function blocking antibodies to PAC1 are undergoing clinical testing for migraine treatment (Amgen) and given our findings, care will need to be taken that such treatments do not impair sensory neuron function in the context of co-existing neuropathy. One means of optimizing delivery to promote neurite outgrowth of small fibres would be to develop small molecule agonists of PAC1 that can be given locally, as has been achieved for PAC1 antagonists (Takasaki *et al.*, 2018) and other growth promoting molecules, such as GDNF (Sidorova *et al.*, 2017).

## Supplementary Material

awaa163_Supplementary_Data

## Abbreviations

CTS =: carpal tunnel syndrome
DEG =: differentially expressed gene
hiPSCd =: human induced pluripotent stem cell-derived
IENFD =: intraepidermal nerve fibre density
PACAP =: pituitary adenylate cyclase activating peptide
