# Corrigendum to: Molecular and cellular correlates of human nerve regeneration: *ADCYAP1*/PACAP enhance nerve outgrowth

**DOI:** 10.1093/brain/awab035

**Published:** 2021-06-02

Georgios Baskozos, Oliver Sandy-Hindmarch, Alex J. Clark, Katherine Windsor, Pall Karlsson, Greg A. Weir, Lucy A. McDermott, Joanna Burchall, Akira Wiberg, Dominic Furniss, David L. H. Bennett and Annina B. Schmid. Molecular and cellular correlates of human nerve regeneration: *ADCYAP1*/PACAP enhance nerve outgrowth. *Brain.* 2020;143:2009–2026. doi:10.1093/brain/awaa163

The authors apologize for errors in Table 3 and Figure 1. These have been corrected.

**Table 3:** Quantitative sensory testing data of patients pre- and 6 months post-surgery as well as healthy controls

	Surgery group			Healthy controls		
	Pre	Post	*P*-value (pre post)	Baseline	*P*-value (HC-pre)	*P*-value (HC-post)
CDT, °C	−5.66 (4.95)	−3.38 (2.33)	**<0.0001**	−2.28 (0.97)	**<0.0001**	**0.033**
WDT, °C	5.25 (3.89)	4.57 (3.09)	0.120	3.01 (1.66)	**0.021**	**0.049**
TSL, °C	11.15 (8.38)	8.74 (5.73)	**0.002**	5.53 (2.89)	**0.002**	**0.023**
MDT, mN	8.46 (19.00)	3.01 (8.90)	**<0.0001**	0.38 (0.26)	**<0.0001**	**0.006**
VDT, x/8	7.45 (0.74)	7.78 (0.54)	**<0.0001**	7.88 (0.25)	**0.015**	0.429
CPT, °C	9.42 (7.00)	11.44 (7.01)	**0.046**	10.72 (7.56)	0.484	0.698
HPT, °C	44.06 (3.94)	43.74 (3.45)	0.481	43.34 (3.98)	0.484	0.664
MPT, mN	180.41 (125.72)	157.27 (103.31)	0.264	153.44 (85.34)	0.657	0.357
MPS, 0–100	0.49 (1.11)	0.30 (0.38)	0.569	0.56 (0.86)	0.690	0.425
PPT, kPa	379.70 (116.08)	397.41 (126.64)	0.221	351.36 (86.22)	0.747	0.452
WUR, ratio	2.53 (2.70)	2.53 (2.24)	0.810	2.04 (1.21)	0.402	0.302

Data are presented as mean (SD) for untransformed data. *P*-values reflect statistics done on untransformed or log transformed data as appropriate comparing pre- and postoperative data (pre post), healthy controls with CTS patients presurgery (HC-pre) and healthy controls with CTS patients postsurgery (HC-post). *P*-values that were significant following Benjamini-Hochberg correction are highlighted in bold. CDT = cold detection threshold; CPT = cold pain threshold; HPT = heat pain threshold; MDT = mechanical detection threshold; MPS = mechanical pain sensitivity; MPT = mechanical pain threshold; PPT = pressure pain threshold; TSL = thermal sensory limen; VDT = vibration detection threshold; WDT = warm detection threshold; WUR = wind-up ratio.

**Figure 1:**
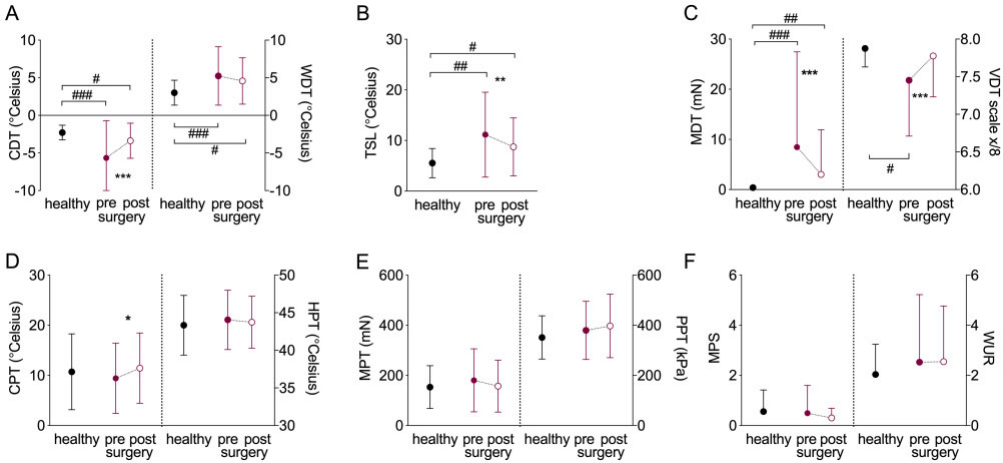
**Recovery of somatosensory function.** Raw quantitative sensory testing data are presented as mean and standard deviations for healthy participants (*n* = 20, filled black circles) and patients with CTS before (filled red circle) and after surgery (*n* = 60, open red circle). (**A**) Cold (CDT) and warm detection thresholds (WDT); (**B**) thermal sensory limen (TSL); (**C**) mechanical (MDT) and vibration detection thresholds (VDT); (**D**) cold (CPT) and heat pain thresholds (HPT); (**E**) mechanical (MPT) and pressure pain thresholds (PPT); (**F**) mechanical pain sensitivity (MPS) and wind-up ratio (WUR). Significant differences for paired/independent *t*-tests after Benjamini Hochberg correction are indicated between groups with ^#^*P* < 0.05, ^##^*P* < 0.01, ^###^*P* < 0.0001 and within groups with *P < 0.05, **P < 0.01, ***P < 0.0001.

