# Supplementary material for: Molecular and cellular correlates of human nerve regeneration: ADCYAP1/PACAP enhance nerve outgrowth
Source: Brain. 2020 Jul 11;143(7):2009–26. doi: 10.1093/brain/awaa163 (PMC7462094; doi:10.1093/brain/awaa163)
Supplement: awaa163_Supplementary_Data [file awaa163_supplementary_data.pdf]

Supplementary Material

Molecular and cellular correlates of human nerve regeneration: *ADCYAP1*/PACAP enhance nerve outgrowth

Supplementary Methods:

Electrodiagnostic tests

Electrodiagnostic tests were performed with an ADVANCE system (Neurometrix, Waltham, MA, USA). Orthodromic sensory latencies and amplitudes were recorded over the digit to wrist segments for the median (index finger), ulnar (little finger) and superficial radial nerve (snuffbox) as previously described (*9*). Compound motor potentials (CMAP) were recorded over the abductor pollicis brevis and adductor digiti minimi by stimulating at the elbow as well as at the wrist. Radial CMAPs (extensor indicis proprius) were recorded by stimulating over the spiral groove. Electrodiagnostic testing was graded according to the scale by Bland[45] as mild (2), moderate (3), severe (4), very severe (5) or extremely severe (6). To determine the presence of a very mild CTS (1), we included two sensitive tests: the presence of a 'double peak' during combined ulnar and median sensory stimulation at the ring finger and recording at the wrist[50] and the presence of prolonged lumbrical to interossei motor latency difference >0.4ms when measured over a fixed distance of 8cm[51]. Hand temperature was standardised to >31 degrees Celsius. During data analysis, absent sensory and motor recordings were replaced with values of zero for amplitudes but excluded from analysis of latencies and nerve conduction velocities to prevent inflated results.

RNA sequencing analysis

RNA-seq data were mapped to the GRC.h.38 Human Genome using the STAR aligner (Dobin *et al.*, 2013) with the ENCODE standard options. Initial quality control was carried out using FastQC, BAM files were sorted, indexed, merged and furthel quality controlled using Samtools (Li et al., 2009).

Read counts were calculated at the gene level using HTSeq (Anders *et al.*, 2015) and the ENSEMBL gene set annotation GRC.h.38.88.

Raw counts were normalised using the effective library size, and for visualisations and associations with phenotypes they were transformed using the variance stabilising transformation (VST) in R using DESeq2 (Love *et al.*, 2014). Library size normalised gene counts were fitted to the negative binomial distribution and hypothesis testing was carried out using the Wald test. P-values were FDR corrected using the Benjamini-Hochberg procedures and the Independent Hypothesis Weighting (IHW) (Ignatiadis *et al.*, 2016). Moderated, i.e. shrunk towards zero, and non-moderated Log 2-fold changes (LFC) were used for hypothesis testing in differential expression (DE) analysis. We considered a gene as significantly DE if it had an adjusted p-value < 0.05 in at least two out of the three hypothesis testing procedures, i.e. moderated LFCs - FDR adjusted p-values, un-moderated LFCs - FDR adjusted p-values, un-moderated LFCs - IHW adjusted p-values.

Gene ontology enrichment for biological processes for DE genes was carried out using topGO (Alexa and Rahnenfuhrer, 2018) and GSEA (Morgan *et al.*, 2017). Hypothesis testing was performed using the weighted Fisher test and the significance cut-off was 0.01. The background gene list consisted of the 18068 genes expressed with > 0 counts in all samples.

Differentiation of human induced pluripotent stem cell (iPSC)-derived sensory neurons

The iPSC line NHDF is a control line derived from a healthy 44-year-old female[69] and the AD2 line from a 51-year-old healthy male. Dermal fibroblasts from these individuals were purchased from Lonza (CC-2511) which were then used for reprogramming to pluripotency. Lonza provide the following ethics statement: 'These cells were isolated from donated human tissue after obtaining permission for their use in research applications by informed consent or legal authorization.' The human iPSCs derived from these fibroblasts were generated as control lines for part of a larger-scale project (Ethics committee: NRES Committee South Central – Berkshire UK, REC 10/H0505/71). The fibroblasts were differentiated to sensory neurons as described previously[12, 13, 70]. In brief, cells were plated at high density following Versene EDTA (ThermoFisher) passaging. Neural induction was initiated in KSR medium (Knockout-DMEM, 15% knockout-serum replacement, 100μM β-mercaptoethanol, 1% nonessential amino acids 1%, Glutamax (ThermoFisher)) by dual SMAD inhibition (SB431542 (Sigma, 10μM) and LDN-193189 (Sigma, 100nM). Three additional small molecules were introduced on day 3: CHIR99021 (Sigma, 3μM), SU5402 (R&D Systems, 10μM) and DAPT (Sigma, 10μM) and dual SMAD inhibitors were withdrawn on day 5. KSR medium was gradually transitioned in 25% increments to neural medium (N2/B27- Neurobasal medium, 2% B27 supplement, 1% N2 supplement, 1% Glutamax, (ThermoFisher)) over an 11-day period. Cells were subsequently dissociated and replated onto glass coverslips in neural medium supplemented with growth factors at 25ng/ml (BDNF; ThermoFisher, NT3, NGF, GDNF; Peprotech). CHIR90221 was included for 4 further days, while SU5402 and DAPT were no longer included at this point. Phenol-free Matrigel (Corning, 1:300 dilution) was included from 25 days onward. Medium

changes were performed twice weekly. Neurons were matured for 27±3 weeks before performing neurite outgrowth assays.

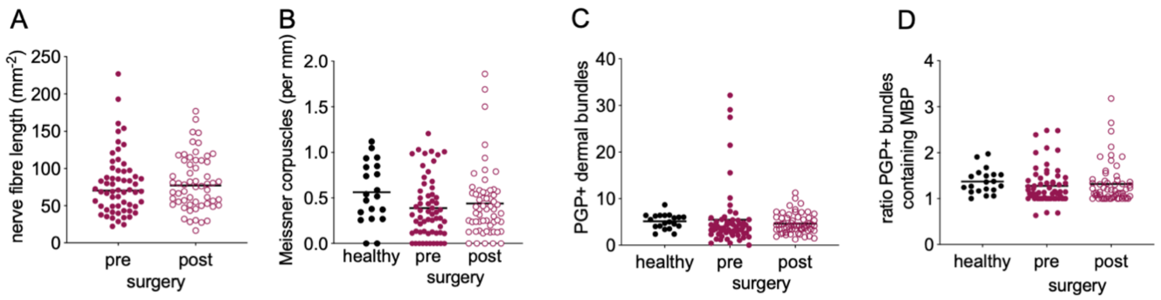

Fig S1: Dermal innervation is comparable between healthy controls and CTS patients and does not change after surgery. **(A)** Subepidermal plexus nerve fibre length **(B)** Meissner corpuscle density **(C)** Protein gene product 9.5 (PGP)+ dermal bundles and **(D)** ratio of PGP+ dermal bundles containing myelin basic protein (MBP).

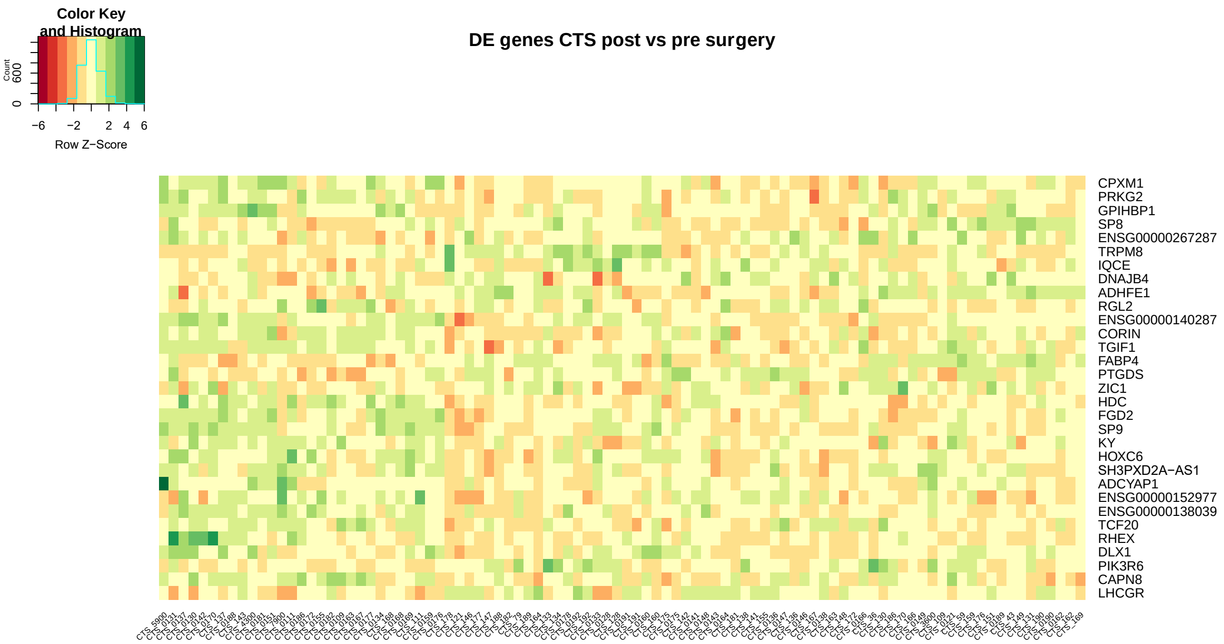

Fig S2: Heatmap of differentially expressed genes identified in the RNA sequencing experiment in human skin (n=47). Heatmap of the relative expression changes based on centered and scaled regularised log2 transformed gene counts. Color key shows the mapping between colours and z-score transformed gene expression.

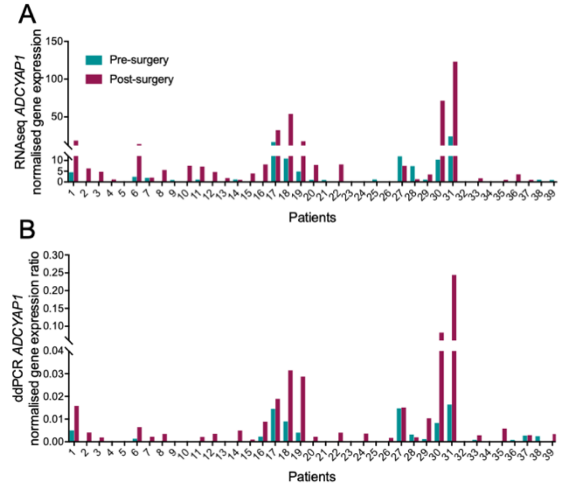

Fig S3: Validation of *ADCYAP1* mRNA expression using droplet digital PCR. **(A)** Normalised gene expression levels of the RNA sequencing align with **(B)** normalised gene expression ratio of the droplet digital PCR experiment (n=39).

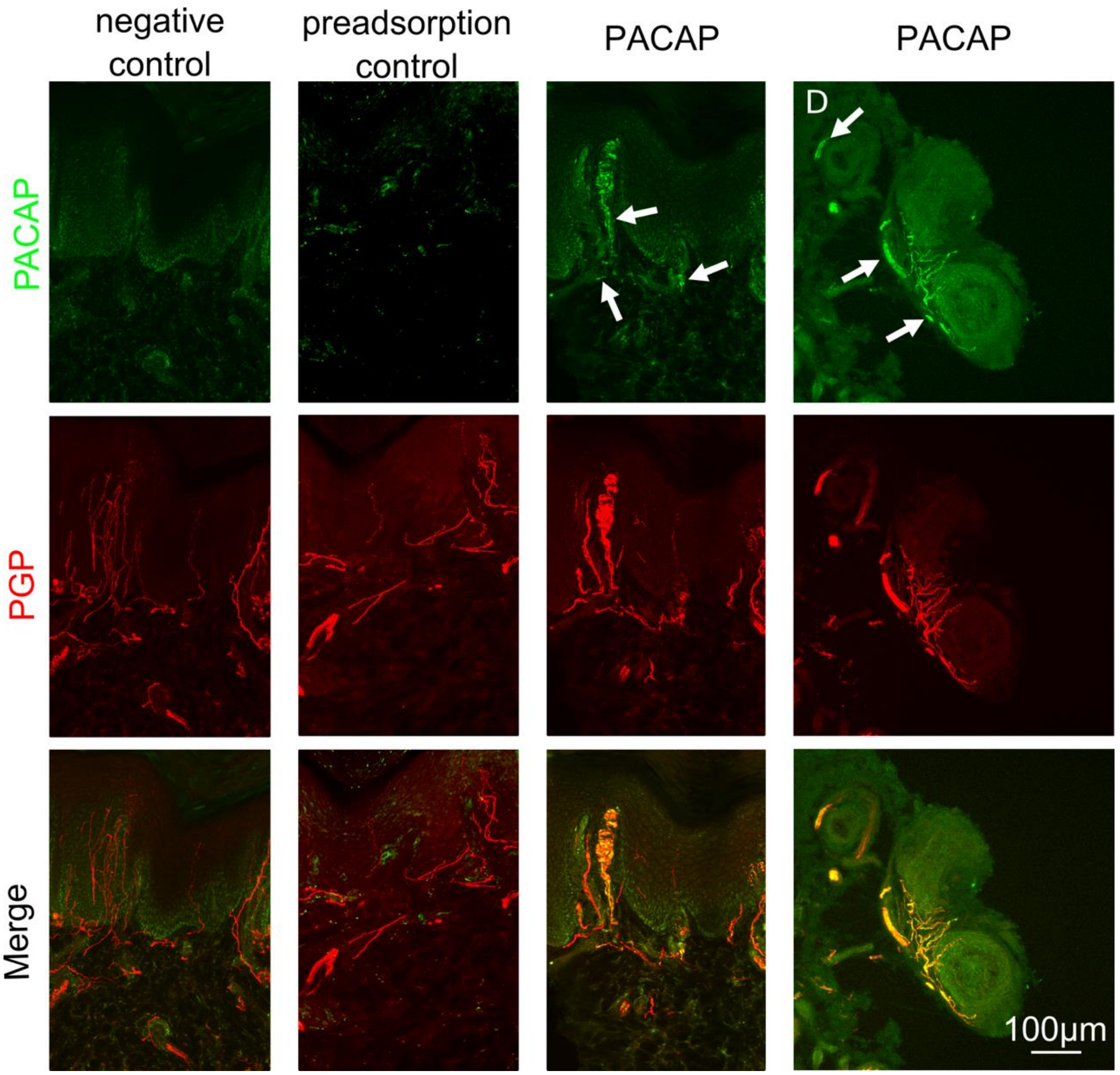

Fig S4: Specificity of PACAP antibody. Human finger skin staining demonstrating specificity of the PACAP antibody with (A) negative control (no primary antibody), (B) preadsorption control (incubation of PACAP antibody with 20μg/ml PACAP protein for 30 minutes before standard staining procedure) and (C) standard PACAP staining. (D) Positive control demonstrating PACAP immunoreactivity within sensory nerve fibers innervating human sweat glands in the dermal layer of the skin. Arrows point to PACAP expression within dermal nerve fibres.

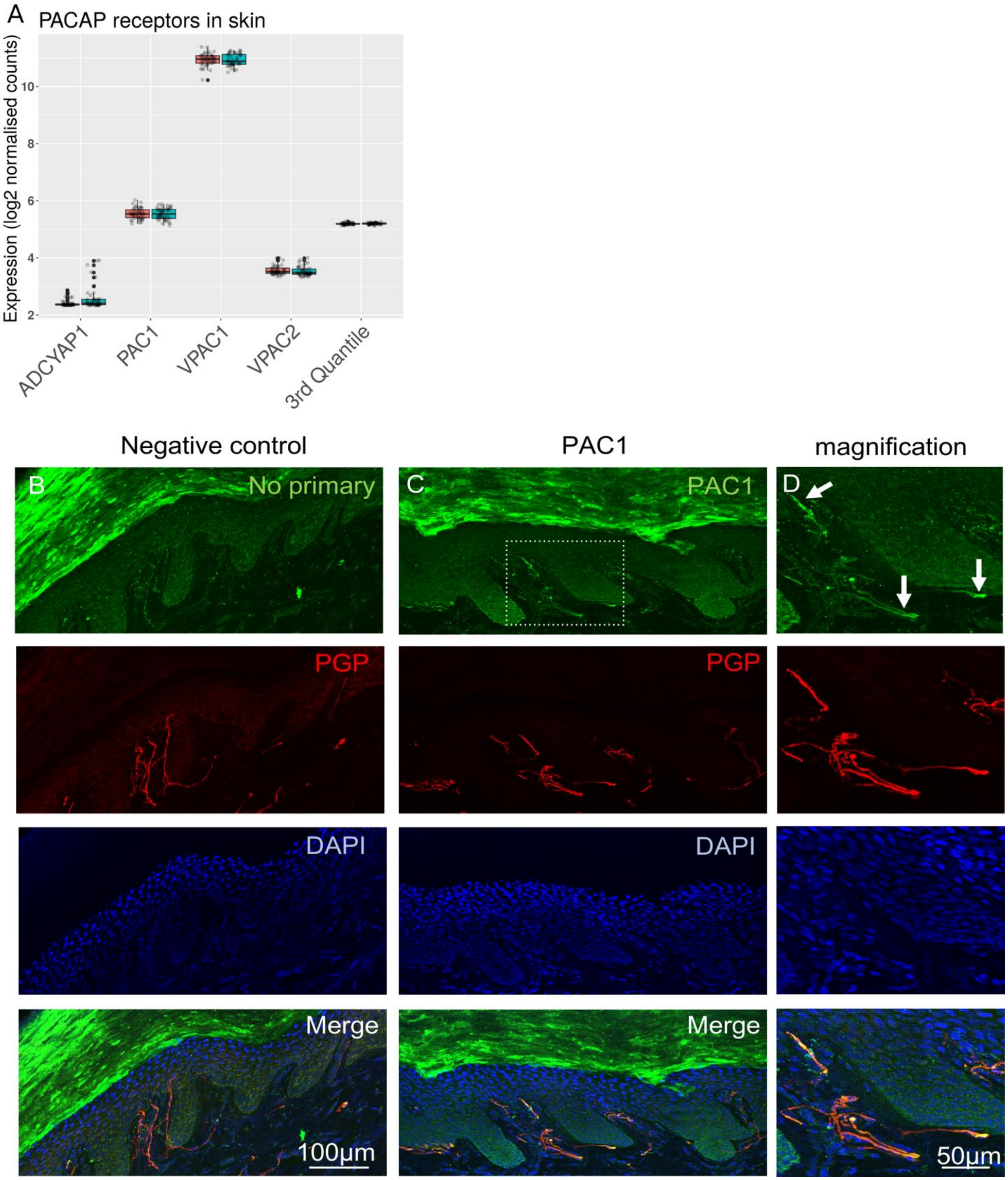

Fig S5: Expression of PAC1 in human skin. (A) mRNA expression levels of *ADCYAP1* and its receptors *PAC1, VPAC1* and *VPAC2* in human skin before (red) and after (blue) carpal tunnel

decompression. The 3rd quantile of all genes is shown for comparison (B-D). Immunostaining for PAC1 revealed low levels of PAC1 protein expression within sensory afferents. (B) negative control by omission of primary antibody (B) PAC1 staining (C) magnified inserts of PAC1 immunoreactivity within sensory neurons (arrows).

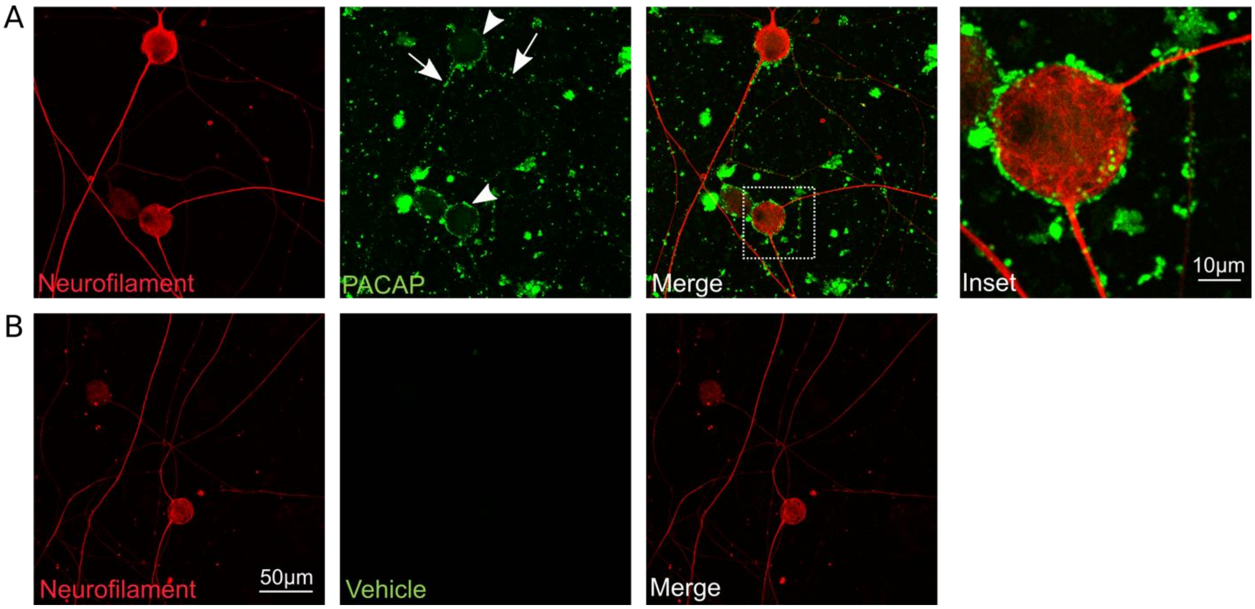

Fig S6: PACAP binding in human induced pluripotent stem cell derived sensory neurons. Cells of the AD2 cell line were replated at ~30weeks old and incubated with biotinylated PACAP (10µMA) or vehicle (B) for 5 days. Aggregated binding of PACAP is apparent around the soma (arrow heads) and axons (arrows) of hiPSCd-sensory neurons.

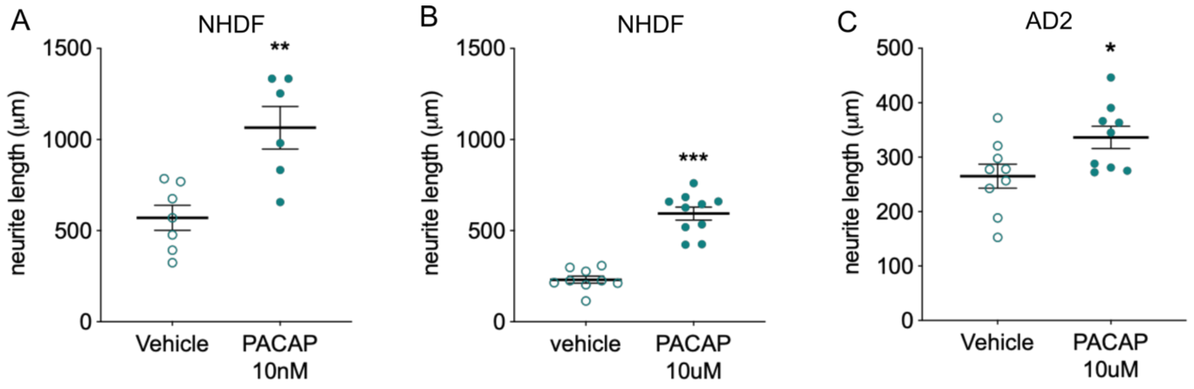

Fig S7: Replication of a regenerative action of PACAP on human induced pluripotent stem cell-derived sensory neurons. PACAP induced enhanced neurite outgrowth in two separate differentiations of the same cell line (NHDF, p<0.003 (A and B)) as well as in a different cell line (AD2, p=0.03). All neurons were 27±3 weeks old at the time of the experiment. Data are presented as mean, SEM and single data points. Please note that the dose of PACAP used in (A) is lower than in (B) and (C).

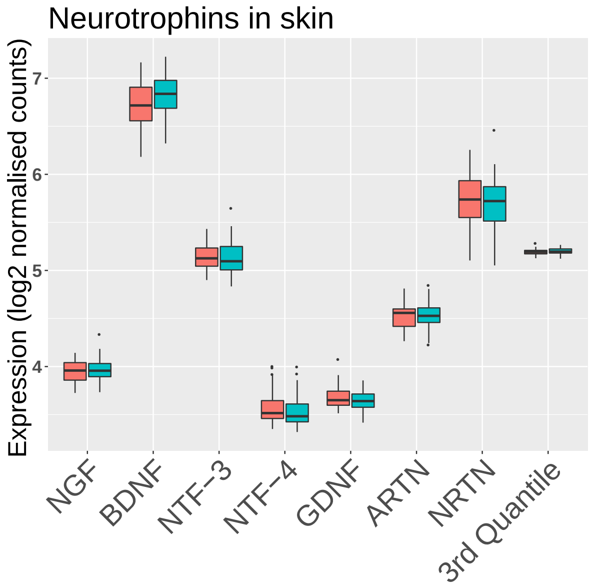

Fig S8: mRNA expression of neurotrophins in the median nerve innervated skin is comparable before (red) and after (blue) carpal tunnel surgery. Data are presented as median and interquartile range and the third quantile of all genes is shown for comparison.

Table S1: Significantly differentially expressed genes in skin post compared to pre surgery

GeneID	baseMean	log2FoldChange	padjBH	padjW	padjBetaPrior	Symbol
ENSG00000141433	8.29462916	1.87901203	0.00696535	0.00641312	0.00010901	ADCYAP1
ENSG00000138039	4.65862691	1.25438865	0.01145755	1	0.01401089	LHCGR
ENSG00000152977	4.89145385	1.11254793	0.00842065	0.01940691	0.03253888	ZIC1
ENSG00000088882	92.5184023	0.92190964	0.00103854	0.00064558	7.93E-06	CPXM1
ENSG00000280113	8.0167541	0.85235048	0.00848225	0.02412323	0.02563488	NA
ENSG00000140287	203.616122	0.72241168	0.00983556	0.0074644	0.03253888	HDC
ENSG00000267453	19.7973425	0.67273101	0.02111925	0.01940691	0.02522041	LINC01835
ENSG00000263961	44.8376836	0.62915302	0.00696535	0.00393004	0.01854591	RHEX
ENSG00000170323	77.6103507	0.62247621	0.05612312	0.02862547	0.02855876	FABP4
ENSG00000276231	35.1351758	0.49145976	0.03282225	0.01705774	0.0435133	PIK3R6
ENSG00000277494	67.4781354	0.44054154	0.00848225	0.00518474	0.00504287	GPIHBP1
ENSG00000107317	135.740333	0.42046293	0.00762641	0.00421831	0.00796676	PTGDS
ENSG00000138669	39.7099245	0.41788473	0.00328312	0.00174962	0.00376299	PRKG2
ENSG00000270605	48.9272349	0.41678525	0.03447244	0.13317736	0.02855876	NA
ENSG00000145244	143.132317	0.30856247	0.03863323	0.01705774	0.03104537	CORIN
ENSG00000147576	120.447618	0.30289174	0.00696535	0.00275544	0.00645172	ADHFE1
ENSG00000197757	287.995304	0.19212071	0.01723099	0.00975572	0.01708509	HOXC6
ENSG00000162616	506.036925	0.17310075	0.0373841	0.07851918	0.03208498	DNAJB4
ENSG00000177426	489.021911	0.13250093	0.01723099	0.02115744	0.01883526	TGIF1
ENSG00000237441	2611.98287	0.10001312	0.03596432	0.01705774	0.03104537	RGL2
ENSG00000144481	17.0057572	-0.7848364	0.00762641	0.01705774	0.00504287	TRPM8
ENSG00000164651	220.844691	-0.5629928	2.77E-09	7.80E-10	9.96E-09	SP8
ENSG00000203697	173.954774	-0.4487044	0.05612312	0.02862547	0.02855876	CAPN8
ENSG00000217236	110.859221	-0.3937448	0.01145755	0.00704249	0.02110566	SP9
ENSG00000267287	80.1845927	-0.3506917	0.03354355	0.01705774	0.03423147	NA
ENSG00000144355	489.75608	-0.3277165	0.00049206	0.0010581	0.00020125	DLX1
ENSG00000174611	165.305979	-0.2733539	0.02060243	0.01705774	0.0241341	KY
ENSG00000146192	551.866588	-0.2174852	0.03596432	0.0496477	0.03953373	FGD2

ENSG00000280693	188.838376	-0.2155644	0.02876924	0.01940691	0.02855876	SH3PXD2A-AS1
ENSG00000100207	1378.70972	-0.1335073	0.02341961	0.0366254	0.02495219	TCF20
ENSG00000106012	714.887208	-0.0974793	0.00593115	0.00491958	0.00497218	IQCE

Table S2: Gene ontology (GO) terms for biological processes identified in skin

GO.ID	Term	Annotated	Significant	Expected	weightFisher
GO:0045766	positive regulation of angiogenesis	117	3	0.2	0.00094
GO:0009954	proximal/distal pattern formation	28	2	0.05	0.00099
GO:0050999	regulation of nitric-oxide synthase activity	35	2	0.06	0.00154
GO:0032611	interleukin-1 beta production	49	2	0.08	0.003
GO:0042632	cholesterol homeostasis	52	2	0.09	0.00338
GO:0006357	regulation of transcription from RNA polymerase II	1626	7	2.72	0.00632
GO:0006810	transport	4071	9	6.82	0.00847
GO:0042089	cytokine biosynthetic process	80	2	0.13	0.00961
GO:0048706	embryonic skeletal system development	99	2	0.17	0.01177
GO:0006821	chloride transport	69	2	0.12	0.01278
GO:0001990	regulation of systemic arterial blood pressure	30	2	0.05	0.0159
GO:0030326	embryonic limb morphogenesis	116	2	0.19	0.01591
GO:0010623	programmed cell death involved in cell development	10	1	0.02	0.01663
GO:0002003	angiotensin maturation	10	1	0.02	0.01663
GO:0071285	cellular response to lithium ion	10	1	0.02	0.01663
GO:0042368	vitamin D biosynthetic process	10	1	0.02	0.01663
GO:0001660	fever generation	10	1	0.02	0.01663
GO:0042033	chemokine biosynthetic process	10	1	0.02	0.01663
GO:0045187	regulation of circadian sleep/wake cycle	10	1	0.02	0.01663
GO:0030656	regulation of vitamin metabolic process	10	1	0.02	0.01663
